# Theoretical Dynamics Modeling of Pitch Motion and Obstacle-Crossing Capability Analysis for Articulated Tracked Vehicles Based on Myriapod Locomotion Mechanism

**DOI:** 10.3390/biomimetics11020121

**Published:** 2026-02-06

**Authors:** Ningyi Li, Xixia Liu, Hongqian Chen, Yu Zhang, Shaoliang Zhang

**Affiliations:** Department of Vehicle Engineering, Army Arms University of PLA, Beijing 100072, China; leeny1992@163.com (N.L.); chenhqaaaf@163.com (H.C.); zhangyuaaaf@163.com (Y.Z.); 18912015572@163.com (S.Z.)

**Keywords:** articulated tracked vehicle, pitch motion, multi-body dynamics, obstacle-crossing capability, track–ground contact pressure, biomimetics, myriapod locomotion

## Abstract

Myriapods achieve remarkable obstacle-crossing capability through inter-segment pitch adjustment and coordinated anterior–posterior propulsion, providing valuable biomimetic inspiration for engineering design. Articulated tracked vehicles, connecting front and rear units via pitch mechanisms, exhibit functional similarity to myriapod body segments. This study develops a comprehensive dynamic model for articulated tracked vehicle pitch motion to reveal its biomimetic connection with myriapod locomotion. A quadratic-function-based non-uniform track–ground contact pressure distribution method with zero-gradient boundary conditions is proposed, effectively eliminating the non-physical negative pressure issue inherent in traditional assumptions. Systematic analyses reveal that the front unit provides primary traction under pitch-up conditions, forming a front-pulling-rear driving mode, while the rear unit dominates under pitch-down and acceleration conditions, forming a rear-pushing-front driving mode. Through pitch attitude adjustment, the maximum surmountable vertical-wall height increased from 263 to 593 mm, representing a 125.45% improvement. This traction distribution pattern closely matches the anterior-guidance and posterior-propulsion mechanism observed in myriapod locomotion. This study quantitatively validates the functional analogy between articulated tracked vehicle pitch dynamics and myriapod inter-segment coordination, providing theoretical foundations for bio-inspired tracked vehicle design.

## 1. Introduction

Myriapods exhibit exceptional terrain adaptability through their unique locomotion mechanisms, providing valuable biomimetic inspiration for engineering design [1,2,3]. Centipedes and millipedes consist of multiple body segments connected by flexible joints that allow dorsoventral pitch adjustment. During obstacle crossing, coordinated inter-segment motion occurs, in which anterior segments lift to negotiate obstacles while posterior segments provide propulsive force, forming a characteristic “front-pulling–rear-pushing” locomotion mode [2,3]. Experimental studies have shown that inter-segment pitch angles can reach 25–35° during obstacle crossing and that distributed propulsion plays a critical role in efficient traversal of complex terrain [4,5]. Inspired by these functional principles, articulated tracked vehicles (ATVs) employ a front–rear articulated structure that enables active pitch adjustment between vehicle units. Compared with conventional single-unit tracked vehicles, ATVs can increase approach and departure angles, improve track–ground conformity over uneven terrain, and significantly enhance obstacle-crossing capability, leading to their widespread application in polar expeditions, rescue operations, emergency response, and military transportation [6,7,8,9,10].

During pitch motion, changes in the vehicle attitude directly affect the load distribution between the road wheels and the track–ground contact pressure distribution, subsequently influencing the traction characteristics and driving stability. When the pitch angle is large, some road wheels may become unloaded, resulting in significant load differences between the grounded road wheels. Therefore, establishing a theoretical model that accurately describes the coupling relationship between the pitch attitude, track–ground contact pressure, and traction performance is of significant theoretical value and engineering significance for enhancing vehicle obstacle-crossing capabilities and motion planning.

Various studies on articulated tracked vehicle pitch motion and obstacle-crossing capabilities have been conducted. Sasaki et al. [11] investigated the relationship between the pitch moment and track–ground contact pressure during slope climbing with the pitch mechanism in a micro-motion state for a prototype dual-unit all-terrain vehicle and proposed a steep-slope crossing method based on a favorable load distribution. Cheng and Dong et al. [12,13,14] simplified the contact relationship between articulated tracked vehicles with pitch attitude and the ground as two road wheels in contact, and they proposed pitch moment calculation methods and vertical-wall obstacle-crossing action sequences based on the static-pitch-attitude conditions. Luo et al. [15] designed an articulated mechanism with an adjustable pitch angle and analyzed the vehicle’s ultimate trench-crossing capability under static conditions. Kim et al. [16] established a coupled simulation model of dual-unit all-terrain vehicles with soft soil and analyzed the dynamic responses under various speed and slope conditions. Li et al. [17,18] employed the ADAMS software to establish a virtual prototype of an articulated deep-sea mining vehicle and conducted simulation studies on the vehicle traversal of steps, trenches, and ridges. Tang et al. [19,20] investigated and compared the trench-crossing capabilities of single-unit and articulated tanks, focusing on separable articulated configurations. Li [21] utilized the RecurDyn software to simulate the trench and cliff crossing processes of articulated tracked vehicles.

The research described above laid the foundation for understanding the pitch motion characteristics of articulated tracked vehicles. However, the developed methods failed to establish the motion coordination relationship between the articulated mechanism and tracks, and they did not clearly reflect the coupling forces and moments between the front and rear units at the pitch mechanism [22]. Furthermore, the coupling mechanism between the pitch motion and traction characteristics was not sufficiently revealed.

In the multibody dynamics modeling of tracked vehicles, Dhir and Sankar [23] established a longitudinal plane dynamics model for tracked vehicles based on the Lagrangian method, employing torsion bar suspension to accurately describe the road wheel motion trajectories. Yamakawa and Watanabe [24] constructed a spatial motion analysis model for tracked vehicles with independent torsion bar suspension, coupling and solving the equations of motion for the unit and the road wheel–swing arm assemblies through constraint equations. Chen et al. [25] considered the interactions between the tracks, road wheels, and unit, establishing a tracked vehicle dynamics model capable of capturing suspension travel variations. Luca et al. [26] developed a reduced-order model for simulating the static and dynamic behaviors of high-speed tracked vehicles, analyzing the effects of the tracks and travel speed on the vehicle static and dynamic mechanical performance. Wu et al. [27] established a unit dynamics model incorporating three-dimensional coupled spatial motion. The model calculation results demonstrated high consistency with commercial software for longitudinal, lateral, and vertical motion simulations, thereby validating the accuracy of the new method. In the research described above, the unit and road wheels were treated as rigid bodies and the suspension system was simplified to a lumped parameter model, achieving an effective balance between computational efficiency and accuracy, as well as providing an important reference for articulated tracked vehicle pitch motion modeling. In parallel with vehicle dynamics modeling, research on innovative suspension architectures has also progressed. For instance, Zhang et al. [28] proposed a sky-hook positive real network-based ISD seat suspension to suppress vibration and enhance ride comfort. Bui et al. [29] developed an adaptive control strategy for semi-active inertial suspension systems, demonstrating the potential of inertia-based suspension architectures in vibration control optimization.

In track–terrain interaction modeling, Bekker [30] established the classical pressure-sinkage model, and Reece and Wong et al. [31,32] proposed modified models accounting for the track shoe width and shape effects. The Janosi–Hanamoto exponential model [33] and the Wong peak softening model [34,35] are applicable for describing the shear stress–displacement relationships in plastic and brittle soils, respectively. Regarding the track–ground contact pressure distributions beneath the tracks, uniform, linear, and sinusoidal distributions, as well as other formulations, have been widely applied [36,37,38,39]. Meanwhile, pull–slip equations, arctangent models, and magic formulas [40,41,42,43,44] are also representative approaches for calculating the track traction forces.

Based on the discussion above, the previous research has the following shortcomings. First, pitch motion theoretical models are predominantly based on static or quasi-static assumptions, lacking systematic analysis of the pitch motion mechanical characteristics under dynamic driving conditions. Second, uniform or linear track–ground contact pressure distribution assumptions may produce non-physical phenomena of negative pressure values under large-pitch-angle conditions, making it difficult to accurately reflect the actual ground contact characteristics. Third, the influence mechanism of the pitch attitude on the front–rear unit traction force allocation remains unclear, constraining the development of cooperative obstacle-crossing control strategies. Meanwhile, in related fields such as multi-legged robotics and articulated vehicle systems, coordinated motion planning and control methods, including gait-free planning strategies [45], model predictive control frameworks [46], geometric path planning [47], and nonlinear MPC approaches [48], have been developed by explicitly considering dynamic constraints. However, the application of such advanced strategies to articulated tracked vehicle pitch control remains limited, primarily due to the lack of a refined dynamics model capable of describing pitch–traction coupling and inter-unit interaction effects. Fourth, existing studies rarely quantitatively compare simulation results with kinematic and dynamic parameters of biological prototypes, limiting the validation of biomimetic design effectiveness.

In our previous work [22], the turning dynamics of articulated tracked vehicles were investigated with explicit consideration of inter-unit coupling forces. Building upon this framework, the present study extends the analysis to pitch motion dynamics during obstacle crossing and examines the associated biomimetic traction coordination mechanisms.

To address these issues, dynamics modeling and an analysis of the pitch motion of articulated tracked vehicles were conducted in this study. A kinematic model of the pitch motion that accounts for the suspension stiffness was established, and geometric constraint relationships between the unit pitch angle, suspension deformation, and track–ground contact length were derived. A non-uniform track–ground contact pressure distribution characterization method based on quadratic functions was proposed, overcoming the limitations of traditional linear distribution assumptions under large-pitch-angle conditions. The influences of the pitch attitude on the road wheel load distribution, track–ground contact pressure, traction force allocation, and inter-unit coupling effects were systematically analyzed, revealing the intrinsic mechanism through which the pitch motion affected the vehicle traction performance and obstacle-crossing capability. Finally, a cooperative obstacle-crossing motion planning method was proposed based on the dynamics model. The contributions of this paper are reflected at three levels: At the biomimetic theory level, the structure–function correspondence between ATVs and myriapod locomotion mechanisms is established, and its consistency is verified through comparison between simulation and biological data; at the methodological level, the proposed quadratic ground pressure distribution model breaks through the limitations of traditional uniform distribution assumptions; and at the engineering application level, the effectiveness of biomimetic pitch attitude adjustment strategy for enhancing obstacle-crossing capability is quantitatively verified through simulation.

The rest of this paper is organized as follows. In Section 2, the kinematic model of the pitch motion for articulated tracked vehicles is established, the vehicle structural parameters and coordinate systems are defined, and the geometric constraint relationships and velocity constraint equations are derived. In Section 3, a pitch motion dynamics model is established, a non-uniform track–ground contact pressure distribution characterization method is proposed, and a track–terrain interaction model and overall vehicle dynamics equations are established. In Section 4, pitch mechanism characteristic analysis, static-pitch-attitude adjustment analysis, and obstacle-crossing capability assessment are presented. In Section 5, the analysis of the vehicle dynamics characteristics under constant-velocity and variable-velocity driving conditions is presented, and a cooperative obstacle-crossing motion planning method is proposed with case validation. Section 6 summarizes the paper and provides future research directions.

## 2. Kinematic Model of Pitch Motion

### 2.1. Vehicle Structure and Biomimetic Correspondence Analysis

To establish the functional mapping relationship between ATVs and myriapods, this section analyzes the correspondence between the two from a structure–function perspective. As shown in Table 1, the hydraulic pitch mechanism corresponds to the dorsoventral joints between myriapod body segments, achieving active pitch adjustment; the torsion bar spring suspension is similar to the compliant leg base structures, enabling passive terrain adaptation; the multi-road-wheel track system corresponds to the multi-leg distributed ground contact method, achieving load distribution and traction force transmission; and the front–rear body coupled drive corresponds to anterior–posterior segment coordination, enabling the “front-pulling-rear-pushing” coordinated propulsion mode. The correspondence summarized in Table 1 is intended to clarify the functional mapping between myriapod locomotion mechanisms and articulated tracked vehicle subsystems, with an emphasis on engineering-relevant roles such as pitch adjustment, load redistribution, and traction coordination, rather than on morphology-level biological replication.

Within this framework, the correspondence discussed here is understood in terms of the consistency of mechanical roles and coordination principles that directly influence obstacle-crossing performance. These functional characteristics are subsequently reflected in the kinematic and dynamic analysis results discussed in Section 4 and Section 5.

**Figure 1:**
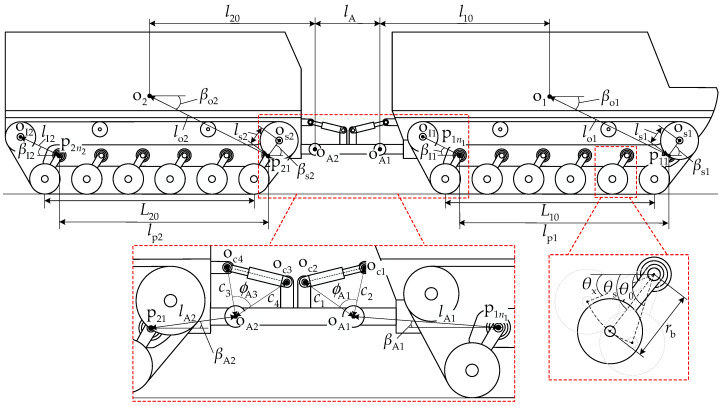
Structure and geometric parameter definitions of the articulated tracked vehicle.

Figure 1 presents the structure and geometric parameter definitions of the articulated tracked vehicle. When the vehicle is stationary on horizontal ground, the initial track–ground contact length is $L_{i0}$, and the longitudinal distance between the front and rear articulation points $o_{A1}$ and $o_{A2}$ is $l_{A}$. For unit i, the center of the k-th road wheel is designated as point $o_{ik}$, with the corresponding torsion bar suspension pivot point as $p_{ik}$. The distance from point $p_{i1}$ to $p_{in_{i}}$ is $l_{pi}$, the distance from the center of mass $o_{i}$ to point $p_{i1}$ is $l_{oi}$, and the angle between the line connecting $o_{i}$ and $p_{i1}$ and the longitudinal axis of unit *i* is $\beta_{oi}$. The distance from the drive sprocket center $o_{si}$ to point $p_{i1}$ is $l_{si}$, and the angle between the line connecting $o_{si}$ and $p_{i1}$ and the longitudinal axis is $\beta_{si}$. The distance from the idler wheel center $o_{Ii}$ to point $p_{in_{i}}$ is $l_{Ii}$, and the angle between the line connecting $o_{Ii}$ and $p_{in_{i}}$ and the longitudinal axis is $\beta_{Ii}$. The angles between the lines connecting $o_{A1}$, $p_{1n_{1}}$ and $o_{A2}$, $p_{21}$ to the longitudinal axes are $\beta_{A1}$ and $\beta_{A2}$, respectively.

#### 2.1.1. Torsion Bar Suspension System

The track system suspension of the articulated tracked vehicle employs torsion bar springs with linear torsional stiffness $k_{t}$. The torsion bar suspension parameters for the front- and rear-unit track systems are identical, as shown in the enlarged local view of the suspension device in Figure 1. The suspension balance arm length is $r_{b}$, the initial position angle between the balance arm and the unit longitudinal axis is $\theta_{0}$, and the angle at maximum limit position relative to the unit longitudinal axis is $\theta_{x}$.

To simplify the analysis, when the vehicle is stationary on horizontal ground, the unit mass $m_{i}$ is assumed to be uniformly distributed longitudinally, all road wheel loads are equal, and all suspension balance arms have the same static rotation angle $\theta_{s}$. $\theta_{s}$ has the following relationship with the initial installation angle $\theta_{0}$:

(1) $$\theta_{0}=\frac{mg}{2n}\frac{r_{b}\cos\theta_{s}}{k_{t}}+\theta_{s},$$

where *n* is the number of road wheels in the track system.

When the pitch attitude of the front and rear units changes, with the pitch angle relative to the road surface being $\eta_{i}$, for the suspension balance arm corresponding to road wheel *k*, the range of $\theta_{ik}$ is

(2) $$\theta_{x}+\eta_{i}\leq \theta_{ik}\leq \theta_{0}+\eta_{i}.$$

For $\theta_{ik}$ satisfying Equation (2), the load on corresponding road wheel *ik* is

(3) $$q_{ik}=\frac{k_{t}(\theta_{0}+\eta_{i}-\theta_{ik})}{r_{b}\cos\theta_{ik}}.$$

where $\theta_{0}+\eta_{i}$ represents the reference angle of the balance arm when unit *i* has pitch angle $\eta_{i}$ relative to the ground.

#### 2.1.2. Pitch Mechanism

**Figure 2:**
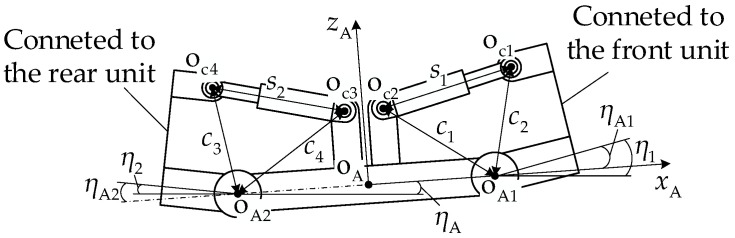
Structure and operating principle of the pitch mechanism.

The pitch mechanism is the most direct biomimetic element, functionally equivalent to the dorsoventral flexion joints between myriapod body segments. Figure 2 illustrates the structure and operating principle of the pitch mechanism. A local coordinate system $x_{A}o_{A}z_{A}$ is established at the midpoint $o_{A}$ of the line connecting $o_{A1}$ and $o_{A2}$. The pitch mechanism drives the front and rear units to rotate about articulation points $o_{A1}$ and $o_{A2}$ through variations in the strokes $s_{1}$ and $s_{2}$ of the front and rear pitch cylinders $o_{c1}o_{c2}$ and $o_{c3}o_{c4}$, respectively. The angles between the unit longitudinal axis and the coordinate system longitudinal axis $x_{A}$ are $\eta_{Ai}$, which have the following formulas:

(4) $$\left\{\begin{array}{l} \eta_{A1}=\phi_{A1}-\arccos\left(\frac{{c_{1}}^{2}+{c_{2}}^{2}-{s_{1}}^{2}}{2c_{1}c_{2}}\right)\\ \eta_{A2}=\phi_{A3}-\arccos\left(\frac{{c_{3}}^{2}+{c_{4}}^{2}-{s_{2}}^{2}}{2c_{3}c_{4}}\right) \end{array}\right..$$

From Equation (4), it can be observed that with a pitch-up attitude, $\eta_{Ai}>0$, and with a pitch-down attitude, $\eta_{Ai}<0$.

The moment arm lengths of the pitch cylinder forces, i.e., the distances from points $o_{A1}$ and $o_{A2}$ to the front and rear cylinders $l_{c1}$ and $l_{c2}$, can be expressed as follows:

(5) $$\left\{\begin{array}{l} l_{c1}=\frac{c_{1}c_{2}}{s_{1}}\sin\left(\phi_{A1}-\eta_{A1}\right)\\ l_{c2}=\frac{c_{3}c_{4}}{s_{2}}\sin\left(\phi_{A3}-\eta_{A2}\right) \end{array}\right..$$

The pitch cylinder force $F_{ci}$ depends on the pressure difference between the rod chamber and rodless chamber and the areas of the two chambers. The maximum working pressure of the cylinder is $p_{c}$, and the maximum cylinder force $F_{ci}$ generated during operation can be expressed as follows:

(6) $$F_{ci}=\left\{\begin{array}{cc} \frac{\pi }{4}\left({d_{c1}}^{2}-{d_{c2}}^{2}\right)p_{c}e_{c}, & \eta_{Ai}\geq 0\\ \frac{\pi }{4}{d_{c1}}^{2}p_{c}e_{c}, & \eta_{Ai}<0 \end{array}\right.,$$

where $d_{c1}$ is the cylinder bore diameter, $d_{c2}$ is the piston rod diameter, and $e_{c}$ is the hydraulic cylinder mechanical efficiency.

The maximum pitch moment $M_{Ai}$ output by the pitch mechanism during operation can be expressed as follows:

(7) $$M_{Ai}=F_{ci}l_{ci}.$$

### 2.2. Coordinate System Definition and Geometric Relationships

**Figure 3:**
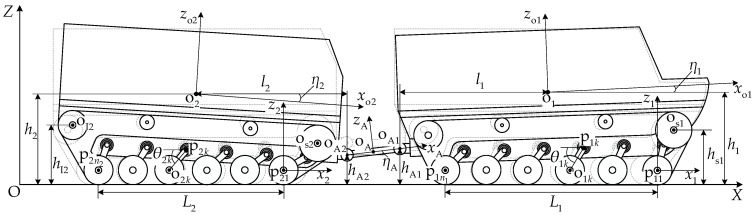
Schematic and geometric constraints of articulated tracked vehicle in pitch-up motion.

Figure 3 illustrates the coordinate system definition and geometric constraint relationships for the vehicle pitch-up attitude. XOZ is the global coordinate system fixed to the ground, $x_{oi}o_{i}z_{oi}$ is the body coordinate system fixed at center of mass $o_{i}$, $x_{i}o_{i1}z_{i}$ is the local coordinate system fixed at the first road wheel center $o_{i1}$, and $x_{A}o_{A}z_{A}$ is the local coordinate system fixed at the midpoint $o_{A}$ of the articulation point $o_{Ai}$ line.

Since there are two degrees of freedom in the pitch mechanism direction, during vehicle pitch motion, there is an angle $\eta_{A}$ between the pitch mechanism and the ground. The relationship between the pitch mechanism angle $\eta_{Ai}$ and the unit pitch angle $\eta_{i}$ is as follows:

(8) $$\eta_{1}-\eta_{A1}=\eta_{A2}-\eta_{2}=\eta_{A}.$$

With a pitch-up attitude, the last road wheel of the front unit and the first road wheel of the rear unit are always load-bearing road wheels. Based on the geometric relationships in Figure 3, the coordinates of key points for the front and rear units in the local coordinate system $x_{i}o_{i1}z_{i}$ are presented in Table 2.

From the coordinates in Table 2, the longitudinal distance $l_{i}$ from the center of mass $o_{i}$ to the articulation point $o_{Ai}$ along the $x_{i}$ axis can be expressed as follows:

(9) $$l_{i}=l_{pi}\cos\eta_{i}+l_{Ai}\cos\left(\beta_{Ai}-\eta_{i}\right)-l_{oi}\cos\left(\beta_{oi}-\eta_{i}\right).$$

The longitudinal distance $l_{ik}$ from the road wheel center $o_{ik}$ to the articulation point $o_{Ai}$ can be expressed as follows:

(10) $$l_{ik}=l_{pi}\frac{n_{i}-k}{n_{i}-1}\cos\eta_{i}+l_{Ai}\cos\left(\beta_{Ai}-\eta_{i}\right)-r_{b}\cos\theta_{ik}.$$

When the vehicle has a pitch angle, at least one of the first and last road wheels is always in contact with the ground, with the wheel center height from the ground equal to the sum of the road wheel radius *r* and the track thickness $h_{t}$, i.e., $r+h_{t}$. From the coordinates of the center of mass $o_{i}$ and the articulation point $o_{Ai}$, the heights $h_{i}$ and $h_{Ai}$ of these two points from the ground can be expressed as follows:

(11) $$l_{ik}=l_{pi}\frac{n_{i}-k}{n_{i}-1}\cos\eta_{i}+l_{Ai}\cos\left(\beta_{Ai}-\eta_{i}\right)-r_{b}\cos\theta_{ik},$$

where $z_{i1}$ and $z_{i6}$ are the normal coordinates of the first and last road wheel centers $o_{ik}$ of unit *i*, respectively.

The drive sprocket center $o_{si}$ height from ground $h_{si}$ can be expressed as follows:

(12) $$h_{si}=r_{b}\sin\theta_{i1}+l_{si}\sin\left(\beta_{si}+\eta_{i}\right)-\min\left\{z_{i1},z_{i6}\right\}+r+h_{t}.$$

The idler wheel center $o_{Ii}$ height from ground $h_{Ii}$ can be expressed as follows:

(13) $$h_{Ii}=r_{b}\sin\theta_{i1}+l_{pi}\sin\eta_{i}+l_{Ii}\sin\left(\beta_{Ii}-\eta_{i}\right)-\min\left\{z_{i1},z_{i6}\right\}+r+h_{t}.$$

When the unit has a pitch-up attitude, as the pitch angle increases, the rear suspension continuously compresses, while the front suspension compression releases until the road wheels become unloaded. When the unit has a pitch-down attitude, as the pitch angle increases, the front suspension continuously compresses while the rear suspension compression releases until the road wheels become unloaded. When unit *i* has $k_{i}$ ($k_{i}$ = 0, 1, …, $n_{i}-1$) unloaded road wheels, the balance arm rotation angles of these $k_{i}$ road wheels satisfy

(14) $$\theta_{ik}=\theta_{0}+\eta_{i}.$$

For the remaining $n_{i}-k_{i}$ grounded road wheels, when $\eta_{i}>0$, there is a $k\in \left[k_{i}+1,\;n_{i}-1\right]$, and when $\eta_{i}<0$, there is a $k\in \left[2,\;n_{i}-k_{i}\right]$. The wheel center points $o_{ik}$ have the same height from the ground, satisfying

(15) $$\left\{\begin{array}{cc} l_{pi}\frac{n_{i}-k}{n_{i}-1}\sin\eta_{i}+r_{b}\sin\theta_{in_{i}}-r_{b}\sin\theta_{ik}=0, & \eta_{i}\geq 0\\ l_{pi}\frac{k-1}{n_{i}-1}\sin\eta_{i}+r_{b}\sin\theta_{ik}-r_{b}\sin\theta_{i1}=0, & \eta_{i}<0 \end{array}\right..$$

The unit pitch angle $\eta_{i}$ can be expressed as follows:

(16) $$\eta_{i}=\left\{\begin{array}{cc} \arcsin\left[\frac{\left(n_{i}-1\right)r_{b}}{\left(n_{i}-k\right)l_{pi}}\left(\sin\theta_{ik}-\sin\theta_{in_{i}}\right)\right], & \eta_{i}\geq 0\\ \arcsin\left[\frac{\left(n_{i}-1\right)r_{b}}{\left(k-1\right)l_{pi}}\left(\sin\theta_{i1}-\sin\theta_{ik}\right)\right], & \eta_{i}<0 \end{array}\right..$$

The track–ground contact length $L_{i}$, which is determined by the track geometry, suspension characteristics, and unit load distribution, can be approximately viewed as the distance between the first and last grounded road wheel centers. When $\eta_{i}>0$, the track–ground contact length $L_{i}$ expression calculated from the grounded road wheels $k_{i}+1$ and $n_{i}$ is

(17) $$L_{i}=l_{pi}\frac{n_{i}-\left(k_{i}+1\right)}{n_{i}-1}\cos\eta_{i}+r_{b}\left(\cos\theta_{in_{1}}-\cos\theta_{i(k_{i}+1)}\right).$$

When $\eta_{i}<0$, the track–ground contact length $L_{i}$ expression calculated from the grounded road wheels 1 and $n_{i}-k_{i}$ is

(18) $$L_{i}=l_{pi}\frac{n_{i}-\left(k_{i}+1\right)}{n_{i}-1}\cos\eta_{i}+r_{b}\left(\cos\theta_{i(n_{i}-k_{i})}-\cos\theta_{i1}\right).$$

There is an angle $\eta_{A}$ between the line connecting articulation points $o_{A1}$ and $o_{A2}$ and the horizontal line. Then,

(19) $$h_{A1}=h_{A2}+l_{A}\sin\eta_{A}.$$

For the grounded road wheels of the front and rear units,

(20) $$\min\left\{z_{11},z_{1n_{1}}\right\}=\min\left\{z_{21},z_{2n_{2}}\right\}.$$

Combining Equations (11), (19), and (20) yields

(21) $$r_{b}\sin\theta_{11}-r_{b}\sin\theta_{21}-l_{p1}\sin\eta_{1}+l_{p2}\sin\eta_{2}+l_{A1}\sin\left(\beta_{A1}-\eta_{1}\right)-l_{A2}\sin\left(\beta_{A2}-\eta_{2}\right)-l_{A}\sin\eta_{A}=0.$$

This equation reflects the relationship between the front- and rear-unit track suspension travel changes and the unit pitch attitude.

### 2.3. Kinematic Constraint Equations

**Figure 4:**
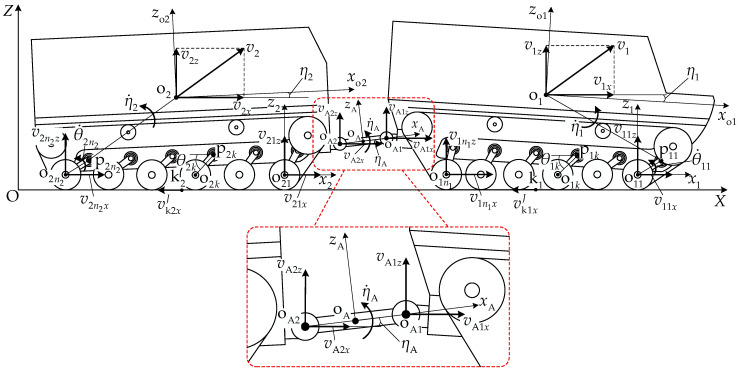
Kinematic relationships of articulated-tracked-vehicle pitch motion.

Figure 4 illustrates the kinematic relationships of articulated-tracked-vehicle pitch motion. The pitch motion of the articulated tracked vehicle includes the planar motion of the vehicle along the *X* direction and changes in the vehicle pitch attitude. The pitch attitude change can be viewed as the combination of the pitch-up unit rotating about the first suspension pivot point and the first suspension balance arm rotating about the first road wheel center, or as the pitch-down unit rotating about the last suspension pivot point and the last suspension balance arm rotating about the last road wheel center.

#### 2.3.1. Unit Velocity Analysis

In local coordinate system $x_{i}o_{i1}z_{i}$, the longitudinal and normal velocity components of the first road wheel center $o_{i1}$ are $v_{i1x}$ and $v_{i1z}$, respectively. The velocity of point $p_{ik}$ can be expressed as follows:

(22) $$\left\{\begin{array}{l} v_{pikx}=v_{i1x}-{\dot{\theta }}_{i1}r_{b}\sin\theta_{i1}-\frac{k-1}{n_{i}-1}l_{pi}{\dot{\eta }}_{i}\sin\eta_{i}\\ v_{pikz}=v_{i1z}+{\dot{\theta }}_{i1}r_{b}\cos\theta_{i1}-\frac{k-1}{n_{i}-1}l_{pi}{\dot{\eta }}_{i}\cos\eta_{i} \end{array}\right..$$

The longitudinal and normal velocity components of the center of mass $o_{i}$ can be expressed, respectively, as follows:

(23) $$\left\{\begin{array}{c} v_{ix}=v_{i1x}-{\dot{\theta }}_{i1}r_{b}\sin\theta_{i1}-{\dot{\eta }}_{i}l_{oi}\sin\eta_{i}\\ v_{iz}=v_{i1z}+{\dot{\theta }}_{i1}r_{b}\cos\theta_{i1}-{\dot{\eta }}_{i}l_{oi}\cos\eta_{i} \end{array}\right..$$

The longitudinal and normal velocity components of articulation point $o_{Ai}$ can be expressed, respectively, as follows:

(24) $$\left\{\begin{array}{c} v_{Aix}=v_{i1x}-{\dot{\theta }}_{i1}r_{b}\sin\theta_{i1}-\left(l_{pi}+l_{Ai}\right){\dot{\eta }}_{i}\sin\eta_{i}\\ v_{Aiz}=v_{i1z}+{\dot{\theta }}_{i1}r_{b}\cos\theta_{i1}-\left(l_{pi}+l_{Ai}\right){\dot{\eta }}_{i}\cos\eta_{i} \end{array}\right..$$

The longitudinal and normal velocity components of the road wheel ik center point $o_{ik}$ can be expressed, respectively, as follows:

(25) $$\left\{\begin{array}{c} v_{ikx}=v_{i1x}-r_{b}\left({\dot{\theta }}_{i1}\sin\theta_{i1}-{\dot{\theta }}_{ik}\sin\theta_{ik}\right)-\frac{k-1}{n_{i}-1}l_{pi}{\dot{\eta }}_{i}\sin\eta_{i}\\ v_{ikz}=v_{i1z}+r_{b}\left({\dot{\theta }}_{i1}\cos\theta_{i1}-{\dot{\theta }}_{ik}\cos\theta_{ik}\right)-\frac{k-1}{n_{i}-1}l_{pi}{\dot{\eta }}_{i}\cos\eta_{i} \end{array}\right..$$

#### 2.3.2. Velocity Constraint Relationships

With a vehicle pitch attitude, the normal velocity component of grounded road wheel centers is zero. When $\eta_{1}>0$ and $\eta_{2}<0$, the last road wheel of the front unit and the first road wheel of the rear unit are always grounded, yielding

(26) $$v_{1n_{i}z}=v_{21z}=0.$$

When $\eta_{1}<0$ and $\eta_{2}>0$, the first road wheel of the front unit and the last road wheel of the rear unit are always grounded, yielding

(27) $$v_{11z}=v_{2n_{i}z}=0.$$

At the articulation point, the velocity relationships are as follows:

(28) $$\left\{\begin{array}{l} v_{A2x}=v_{A1x}-l_{A}{\dot{\eta }}_{A}\sin\eta_{A}\\ v_{A2z}=v_{A1z}+l_{A}{\dot{\eta }}_{A}\cos\eta_{A} \end{array}\right..$$

Combining Equations (24) and (28) yields

(29) $$\left\{\begin{array}{l} v_{11x}-v_{21x}=r_{b}\left({\dot{\theta }}_{11}\sin\theta_{11}+{\dot{\theta }}_{21}\sin\theta_{21}\right)+\left(l_{p1}+l_{A1}\right){\dot{\eta }}_{1}\sin\eta_{1}-\left(l_{p2}+l_{A2}\right){\dot{\eta }}_{2}\sin\eta_{2}+l_{A}{\dot{\eta }}_{A}\sin\eta_{A}\\ v_{11z}-v_{21z}=-r_{b}\left({\dot{\theta }}_{11}\cos\theta_{11}-{\dot{\theta }}_{21}\cos\theta_{21}\right)+\left(l_{p1}+l_{A1}\right){\dot{\eta }}_{1}\cos\eta_{1}-\left(l_{p2}+l_{A2}\right){\dot{\eta }}_{2}\cos\eta_{2}-l_{A}{\dot{\eta }}_{A}\cos\eta_{A} \end{array}\right..$$

#### 2.3.3. Track Motion Analysis

The longitudinal component of the transport velocity at point $k_{i}$ on the track–ground contact segment of unit *i* is $v_{kix}$, which can be expressed as follows:

(30) $$\left\{\begin{array}{l} v_{kix}=v_{in_{i}x},\eta_{i}\geq 0\\ v_{kix}=v_{i1x},\eta_{i}<0 \end{array}\right..$$

Given the track wrapping velocity relative to the unit $v_{di}=r\omega_{i}$, the longitudinal sliding velocity $v_{kix}^{J}$ at point $k_{i}$ is

(31) $$v_{kix}^{J}=v_{kix}-v_{di}=\left\{\begin{array}{c} v_{in_{i}x}-r\omega_{i},\eta_{i}\geq 0\\ v_{i1x}-r\omega_{i},\eta_{i}<0 \end{array}\right..$$

## 3. Pitch Motion Dynamics Modeling

### 3.1. Track–Terrain Interaction

#### 3.1.1. Road Wheel Load Distribution

**Figure 5:**
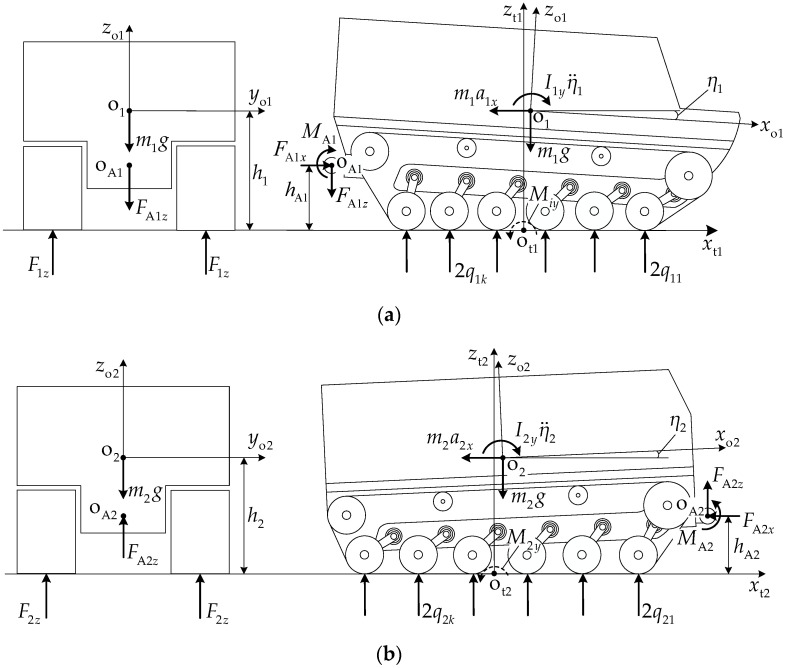
Track system load distribution schematic: (**a**) front unit and (**b**) rear unit.

Figure 5 presents the load distribution schematic of the articulated-tracked-vehicle track system. The single-side track system load $F_{iz}$ is jointly determined by the unit mass $m_{i}$ and the normal component $F_{Aiz}$ of the pitch mechanism coupling force acting at articulation point $o_{Ai}$, while the road wheel load $q_{ik}$ is simultaneously affected by the inertial force $m_{i}a_{ix}$, inertial moment $I_{iy}{\ddot{\eta }}_{i}$, articulation point $o_{Ai}$, longitudinal force $F_{Aix}$, normal force $F_{Aiz}$, and pitch moment $M_{Ai}$. From the normal force equilibrium condition,

(32) $$F_{iz}=\sum_{k=1}^{n_{i}}q_{ik}=\frac{1}{2}\left(m_{i}g\pm F_{Aiz}\right).$$

A local coordinate system $x_{ti}o_{ti}z_{ti}$ is established at the midpoint $o_{ti}$ of the track–ground contact segment. The moment $M_{iy}$ of load $q_{ik}$ about point $o_{ti}$ can be expressed as follows:

(33) $$M_{iy}=\sum_{k=1}^{n_{i}}q_{ik}x_{tik},$$

where $x_{tik}$ is the x-coordinate of wheel center point $o_{ik}$ in coordinate system $x_{ti}o_{ti}z_{ti}$. Based on the coordinates in Table 2,

(34) $$x_{tik}=r_{b}\left(\cos\theta_{i1}-\cos\theta_{ik}\right)-l_{si}\frac{k-1}{n_{i}-1}\cos\eta_{i}+\frac{L_{i}}{2}.$$

From the moment equilibrium condition of unit *i* about point $o_{ti}$,

(35) $$M_{iy}=\frac{1}{2}\left[m_{i}gx_{\mathrm{to}i}-m_{i}a_{ix}h_{i}+I_{iy}{\ddot{\eta }}_{i}\pm \left(F_{Aix}h_{Ai}-F_{Aiz}x_{\mathrm{tA}i}\right)-M_{Ai}\right],$$

where $I_{iy}$ is the moment of inertia of unit *i*, ${\ddot{\eta }}_{i}$ is the pitch angular acceleration of unit *i*, and $x_{toi}$ and $x_{tAi}$ are the longitudinal coordinates of center of mass point $o_{i}$ and articulation point $o_{Ai}$, respectively, in coordinate system $x_{ti}o_{ti}z_{ti}$. Based on the coordinates in Table 2,

(36) $$\left\{\begin{array}{l} x_{toi}=r_{b}\cos\theta_{i1}-l_{gi}\cos\left(\beta_{gi}-\eta_{i}\right)+\frac{L_{i}}{2}\\ x_{tAi}=r_{b}\cos\theta_{i1}-l_{si}\cos\eta_{i}-l_{Ai}\cos\left(\beta_{Ai}-\eta_{i}\right)+\frac{L_{i}}{2} \end{array}\right..$$

#### 3.1.2. Track–Ground Contact Pressure Distribution

The following assumptions are made for the normal pressure distribution in the track–ground contact segment.

(1) The pressure is continuously distributed within the track–ground contact segment.
(2) By neglecting the influence of the track width b, the normal pressure is uniformly distributed laterally and varies only along the track longitudinal direction.

**Figure 6:**
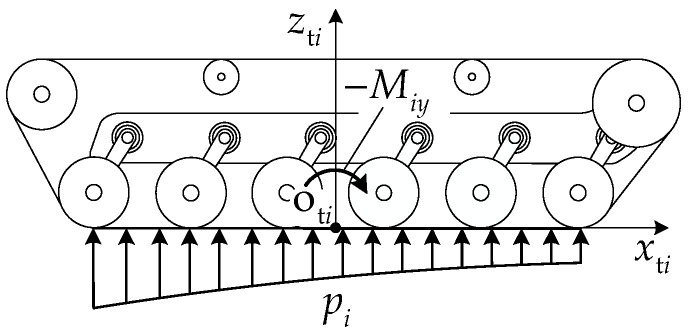
Normal pressure distribution schematic of track–ground contact segment.

Figure 6 presents the normal pressure distribution schematic of the track–ground contact segment. From the normal force equilibrium condition and moment equilibrium condition about point $o_{ti}$, the pressure function $p_{i}\left(x_{ti}\right)$ satisfies

(37) $$\left\{\begin{array}{l} \int_{-L_{i}/2}^{L_{i}/2}bp_{i}dx_{ti}=F_{iz}\\ \int_{-L_{i}/2}^{L_{i}/2}bp_{i}x_{ti}dx_{ti}=M_{iy} \end{array}\right..$$

With a pitch attitude of the articulated tracked vehicle, the load difference $\Delta q_{ik}$ between road wheels can be expressed as follows:

(38) $$\Delta q_{ik}=q_{ik}-\min\left\{q_{i1},q_{in_{i}}\right\}.$$

When the load differences between road wheels are large, the classical linear normal pressure distribution assumption results in negative pressure values beneath the lower-load road wheels. In this work, a quadratic distribution is adopted to characterize the ground contact pressure. The ground contact pressure is divided into the sum of two parts: one part corresponds to the uniform pressure under the action of $\min\left\{n_{i}q_{i1},\;n_{i}q_{in_{i}}\right\}$ and the other part corresponds to the non-uniform pressure distribution caused by the sum of $\Delta q_{ik}$. It is assumed that the normal pressure $p_{i}\left(x_{ti}\right)$ takes the following form:

(39) $$p_{i}\left(x_{ti}\right)=u_{1}{\left(x_{ti}\right)}^{2}+u_{2}\left(x_{ti}\right)+u_{3}.$$

where the pressure distribution function $p_{i}\left(x_{ti}\right)$ contains three undetermined coefficients $u_{1}$, $u_{2}$, and $u_{3}$. The terms containing $x_{ti}$ represent the pressure variation caused by load non-uniformity, while $u_{3}$ represents the uniform pressure under the action of $\min\left\{n_{i}q_{i1},\;n_{i}q_{in_{i}}\right\}$, which can be expressed as follows:

(40) $$u_{3}=\frac{1}{bL_{i}}\left(F_{iz}-\sum_{k=1}^{n_{i}}\Delta q_{ik}\right)=\left\{\begin{array}{l} \begin{array}{cc} \frac{1}{bL_{i}}n_{i}q_{i1}, & \eta_{i}\geq 0 \end{array}\\ \begin{array}{cc} \frac{1}{bL_{i}}n_{i}q_{in_{i}}, & \eta_{i}<0 \end{array} \end{array}\right..$$

By combining Equations (37) and (40), $u_{1}$, $u_{2}$, and $u_{3}$ can be solved for. Substituting them into Equation (38) yields

(41) $$p_{i}\left(x_{ti}\right)=\frac{12}{b{L_{i}}^{3}}\sum_{k=1}^{n_{i}}\Delta q_{ik}{x_{ti}}^{2}+\frac{12}{b{L_{i}}^{3}}M_{iy}x_{ti}+\frac{1}{bL_{i}}\left(F_{iz}-\sum_{k=1}^{n_{i}}\Delta q_{ik}\right).$$

**Figure 7:**
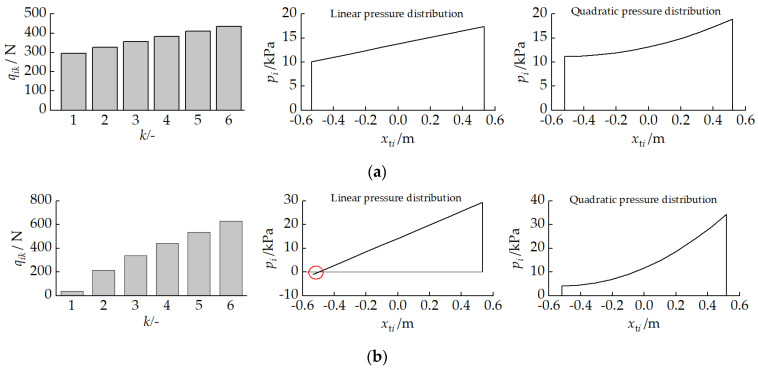
Comparison of linear and quadratic track–ground contact pressure distribution models with different pitch attitudes: (**a**) $\eta_{i}=-1{}^{\circ}$ and (**b**) $\eta_{i}=-4{}^{\circ}$.

Figure 7 compares the linear and quadratic track–ground contact pressure distribution models under different pitch attitudes. When the pitch angle is small, both models produce physically admissible pressure distributions. As the pitch angle increases and load non-uniformity becomes pronounced, the linear distribution yields negative normal pressure in the low-load region of the contact segment, which is highlighted by red circles and is physically inadmissible for track–ground contact. In contrast, the quadratic distribution maintains non-negative pressure over the entire contact length while satisfying the same force and moment equilibrium constraints, indicating that it is suitable for track–ground contact pressure modeling under large pitch-angle conditions.

#### 3.1.3. Shear Displacement and Shear Force

**Figure 8:**
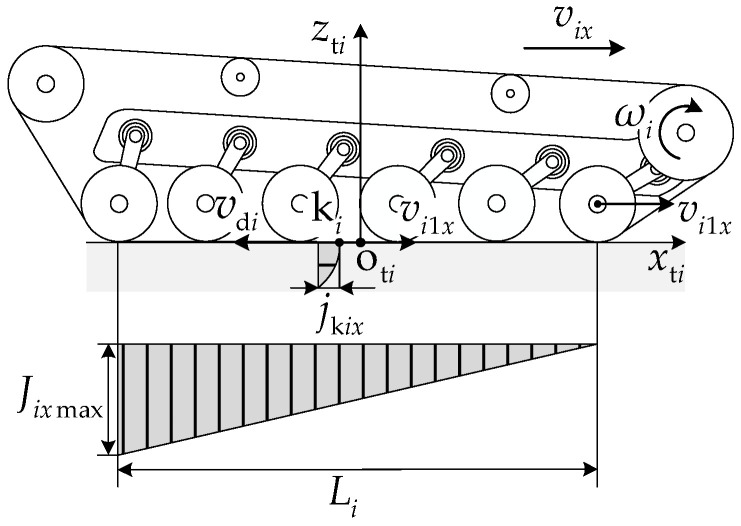
Track shear displacement schematic with a pitch-down attitude.

Figure 8 presents the track shear displacement schematic for a unit pitch-down attitude. The shear displacement increases linearly along the track longitudinal direction from front to rear. The time $t_{i}$ for point $k_{i}$ on the track–ground contact segment of unit *i* to move from position $L_{i}/2$ at the front of the track to position $x_{ti}$ can be expressed as follows:

(42) $$t_{i}=\int_{0}^{t_{i}}dt=\int_{x_{ti}}^{L_{i}/2}\frac{dx_{ti}}{v_{di}}=\frac{L_{i}/2-x_{ti}}{r\omega_{i}}.$$

The shear displacement produced by the track of unit *i* along the *X* direction in the global coordinate system is as follows:

(43) $$J_{Xi}=\int_{0}^{t_{i}}v_{kix}^{J}dt_{i}=\left(L_{i}/2-x_{ti}\right)\left(1-\frac{v_{i1x}}{r\omega_{i}}\right).$$

According to the track–soil shear stress interaction model proposed by Wong et al. [35], when the vehicle travels on hard ground and paved surfaces without considering the adhesion effect between the track and soil, the interaction force between the track and ground can be characterized through the shear displacement–shear stress relationship. Under firm ground conditions, the relationship between the ground shear stress and the normal pressure is as follows:

(44) $$\tau_{i}=p_{i}\mu \left(1-e^{-J_{i}/K}\right),$$

where μ is the friction coefficient between the track and ground, and K is the shear displacement parameter. In this study, μ=0.68 and K=0.12.

The longitudinal shear force $F_{ix}^{J}$ experienced by the track–ground contact segment is always opposite to the sliding velocity $v_{kix}^{J}$ direction, with the expression

(45) $$F_{ix}^{J}=\int_{-L_{i}/2}^{L_{i}/2}bp_{i}\mu \left(1-e^{-J_{iX}/K}\right)dx_{ti}.$$

### 3.2. Track System Driving Mechanics Modeling

#### 3.2.1. Internal Rolling Resistance

**Figure 9:**
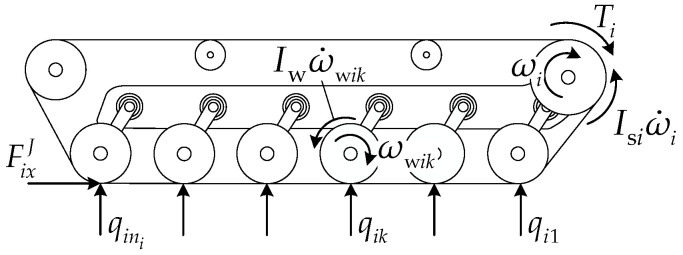
Track assembly driving mechanics analysis schematic.

Figure 9 presents the schematic of the track assembly driving mechanics analysis. The track is subjected to a longitudinal shear force from the ground, a drive sprocket driving force, and road wheel rolling resistance. The drive sprocket output torque $T_{i}$ can be calculated by the following equation:

(46) $$T_{i}=F_{ix}^{J}r+I_{si}{\dot{\omega }}_{i}+\sum_{k=1}^{n_{i}}\left[I_{w}{\dot{\omega }}_{wik}+q_{ik}\left(f_{0}+f_{s}v_{ik}\right)r\right],$$

where $I_{si}$ is the drive sprocket moment of inertia, $I_{w}$ is the road wheel moment of inertia, $\omega_{wik}$ is the rolling angular velocity of the *k*-th road wheel, $v_{ik}$ is the rolling linear velocity of the *k*-th road wheel, and $f_{0}$ and $f_{s}$ are empirical coefficients, taken as 0.03 and 0.00015, respectively.

#### 3.2.2. External Motion Resistance

The track external motion resistance $F_{i}^{f}$ is calculated as follows:

(47) $$F_{i}^{f}=F_{iz}f_{r}=\sum_{k=1}^{n_{i}}q_{ik}f_{r},$$

where $f_{r}$ is the track external motion resistance coefficient. In this study, $f_{r}=0.025$.

#### 3.2.3. Drive Mode

During pitch motion of articulated tracked vehicles, the left and right track motion patterns are identical. For electrically driven articulated tracked vehicles, a power distribution method with the same driving speed for all four tracks is typically adopted, satisfying

(48) $$\omega_{1}=\omega_{2}.$$

### 3.3. Pitch Dynamics Equations

**Figure 10:**
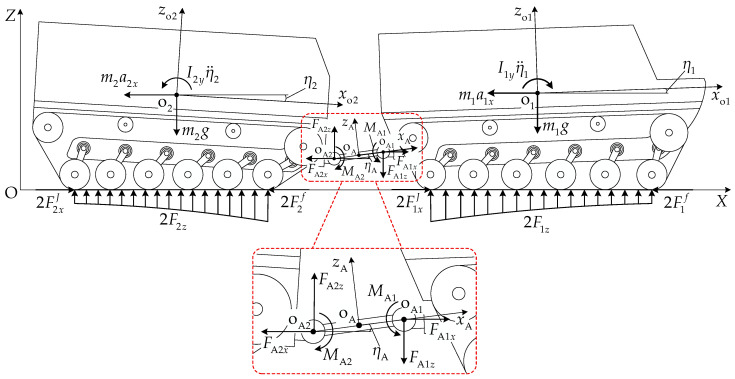
Force analysis schematic of articulated tracked vehicle in pitch-up motion.

The force conditions for vehicle pitch-up and pitch-down motions are similar, with the main difference being the changes in the relative geometric relationships of key points. Figure 10 presents the force analysis schematic of the articulated tracked vehicle in pitch-up motion. From the longitudinal and normal force equilibrium at the pitch mechanism and the moment equilibrium about articulation point $o_{A2}$, the following relations are satisfied:

(49) $$\left\{\begin{array}{l} F_{A1x}-F_{A2x}=0\\ F_{A1z}-F_{A2z}=0\\ M_{A1}-M_{A2}-\left(F_{A1z}\cos\eta_{A}+F_{A1x}\sin\eta_{A}\right)l_{A}=0 \end{array}\right..$$

From the longitudinal and normal mechanical equilibrium conditions and the moment equilibrium condition about articulation point $o_{Ai}$ for the front and rear units, the following relations are satisfied:

(50) $$\left\{\begin{array}{l} 2F_{i}^{J}-2F_{i}^{f}-m_{i}a_{i}\pm F_{Aix}=0\\ 2F_{i}^{J}-2F_{i}^{f}-m_{i}a_{i}\pm F_{Aiz}=0\\ m_{i}g{l^{\prime }}_{i}-2\sum_{k=1}^{n_{i}}q_{ik}l_{ik}\mp \left(2F_{i}^{J}-2F_{i}^{f}\right){h^{\prime }}_{Ai}\mp m_{i}a_{i}\left({h^{\prime }}_{i}-{h^{\prime }}_{Ai}\right)+I_{yi}{\ddot{\eta }}_{i}-M_{Ai}=0 \end{array}\right..$$

Combining Equations (49) and (50) yields the overall vehicle dynamics equations:

(51) $$\left\{\begin{array}{l} 2F_{1}^{J}+2F_{2}^{J}-2F_{1}^{f}-2F_{2}^{f}-m_{1}a_{1}-m_{2}a_{2}=0\\ 2F_{1z}+2F_{2z}-m_{1}g-m_{2}g=0\\ m_{1}g\left(l_{1}+l_{A}\cos\eta_{A}\right)-2\sum_{k=1}^{n_{1}}q_{1k}\left(l_{1k}+l_{A}\cos\eta_{A}\right)-2\left(F_{1}^{J}-F_{1}^{f}\right)\left(h_{A1}-l_{A}\sin\eta_{A}\right)-m_{1}a_{1}\left(h_{1}-h_{A1}-l_{A}\sin\eta_{A}\right)\\ -m_{2}gl_{2}+2\sum_{k=1}^{n_{2}}q_{2k}l_{2k}-2\left(F_{2}^{J}-F_{2}^{f}\right)h_{A2}-m_{2}a_{2}\left(h_{2}-h_{A2}\right)+I_{y1}{\ddot{\eta }}_{1}-I_{y2}{\ddot{\eta }}_{2}=0 \end{array}\right..$$

## 4. Numerical Calculation and Results Analysis

### 4.1. Vehicle Parameters

A specific type of articulated tracked vehicle was selected as the study object, and computational analysis was performed on its pitch mechanism working capability and kinematic and mechanical relationships under static-pitch-attitude conditions. Table 3 presents the main parameters of the articulated tracked vehicle and the ground, and Table 4 presents the main parameters of the pitch mechanism.

### 4.2. Pitch Mechanism Characteristic Analysis

**Figure 11:**
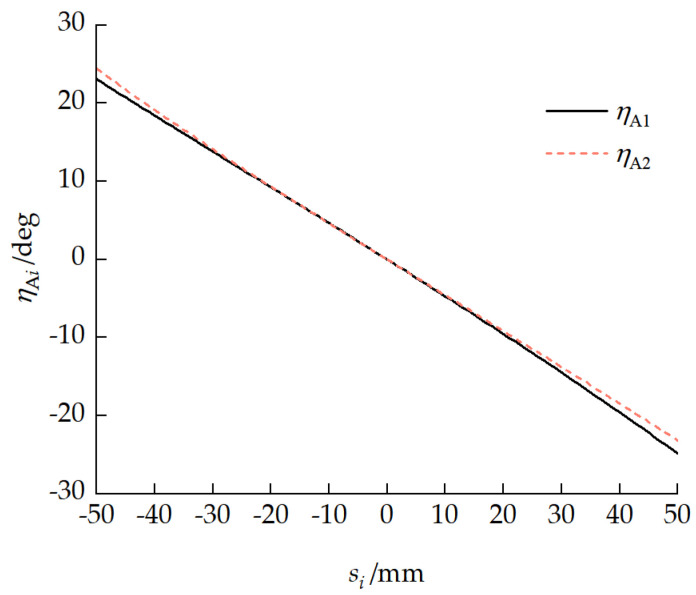
Motion characteristic curves of the pitch mechanism.

**Figure 12:**
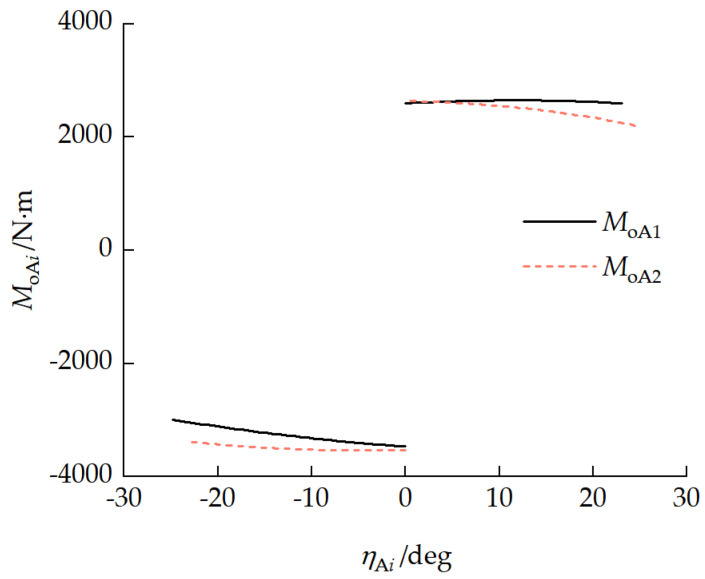
Moment characteristic curves of the pitch mechanism.

Figure 11 shows the motion characteristic curves of the pitch mechanism. The maximum pitch angle that could be produced when the front and rear pitch cylinders retracted was 47.51°, and the maximum pitch angle that could be produced when the cylinders extended was 48.03°. Figure 12 presents the moment characteristic curves of the pitch mechanism. The maximum pitch-up moment range that could be provided when the pitch cylinder retracted was 2206.50 to 2650.72 N·m. The maximum pitch-down moment range that could be provided when the pitch cylinder was extended was −2922.93 to −3537.10 N·m.

### 4.3. Static-Pitch-Attitude Adjustment Analysis

The vehicle performed stationary pitch attitude adjustment with the pitch mechanism cylinder extension/retraction amount $\Delta s_{i}$ as the input. Computational analysis was conducted on the vehicle pitch attitude, inter-unit mechanical interaction relationships, and track system–ground contact characteristics.

#### 4.3.1. Vehicle Pitch Attitude Characteristics

**Figure 13:**
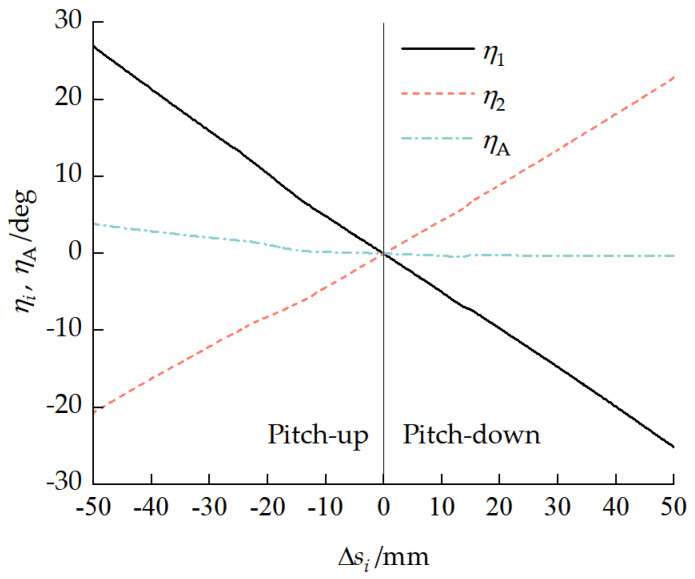
Relationships between unit pitch angle $\eta_{i}$ and pitch mechanism deflection angle $\eta_{A}$ and cylinder extension/retraction amount $\Delta s_{i}$.

The vehicle pitch attitude depends on each unit pitch angle $\eta_{i}$ and pitch mechanism deflection angle $\eta_{A}$. Figure 13 presents the relationships between the unit pitch angle $\eta_{i}$ and pitch mechanism deflection angle $\eta_{A}$ and the cylinder extension/retraction amount $\Delta s_{i}$. When the cylinder retracted, the vehicle assumed a pitch-up attitude. At the maximum pitch-up attitude, the front- and rear-unit pitch angles $\eta_{1}$ and $\eta_{2}$ were 26.87° and −20.65°, respectively. When the cylinder extended, the vehicle assumed a pitch-down attitude. At the maximum pitch-down attitude, the front- and rear-unit pitch angles $\eta_{1}$ and $\eta_{2}$ were −25.19° and 22.84°, respectively. At the maximum vehicle pitch-up attitude, there was a maximum deflection angle $\eta_{\mathrm{Amax}}=3.79{}^{\circ}$ between the pitch mechanism and the ground.

#### 4.3.2. Inter-Unit Coupling Forces

**Figure 14:**
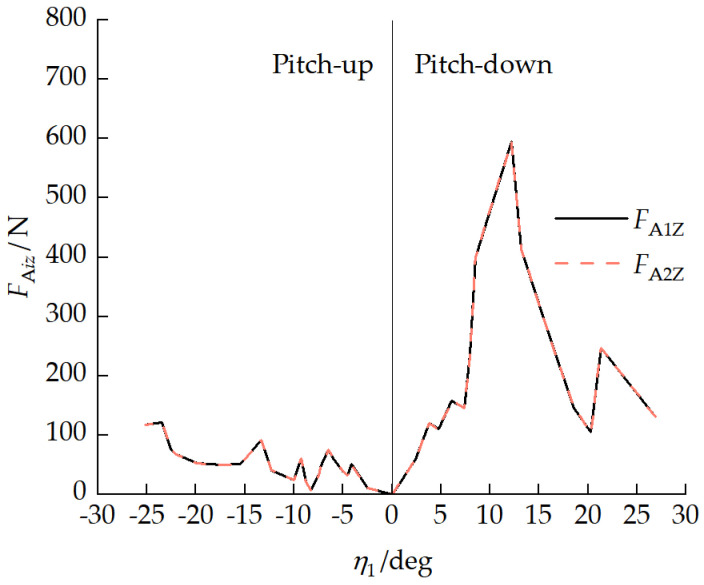
Relationship between normal force $F_{Aiz}$ and front-unit pitch angle $\eta_{1}$.

When the vehicle maintained a stationary pitch attitude, the force and moment interaction between the front and rear units manifested as the longitudinal component $F_{Aix}$, normal component $F_{Aiz}$, and pitch moment $M_{Ai}$ of the pitch mechanism coupling force with a static pitch attitude, $F_{Aix}=0$. Figure 14 shows the relationship between the normal force $F_{Aiz}$ and the front-unit pitch angle $\eta_{1}$. With a vehicle pitch attitude, $F_{Aiz}>0$, indicating that the rear unit transferred load to the front unit through the pitch mechanism, and the front-unit track bore more of a mass load. When the front-unit pitch angle increased, the front-unit suspension balance arm first reached its limit.

**Figure 15:**
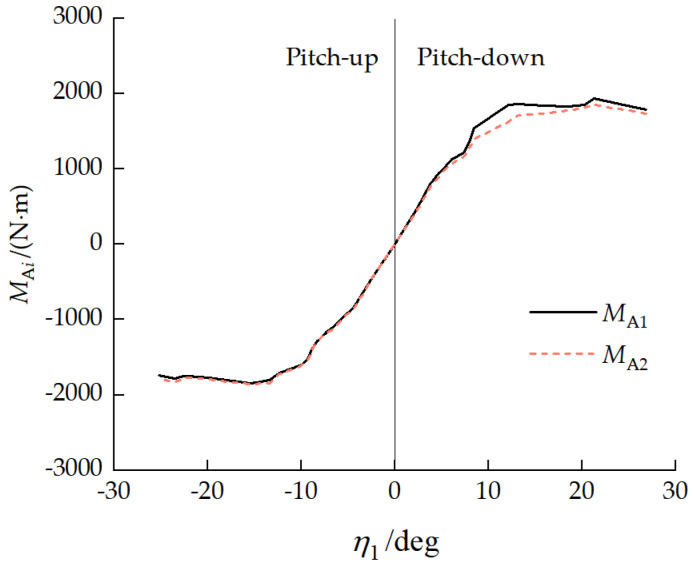
Relationship between pitch moment $M_{Ai}$ and front-unit pitch angle $\eta_{1}$.

Figure 15 presents the relationship between the pitch moment $M_{Ai}$ and the front-unit pitch angle $\eta_{1}$. Since the normal force $F_{Aiz}$ transferred a load to the front unit, it manifested as $M_{A1}\geq M_{A2}$. In the range of $-15{}^{\circ}<\eta_{1}<20{}^{\circ}$, the pitch moment requirement increased rapidly as the front-unit pitch angle increased. When the front-unit pitch angle $\eta_{1}=21.30{}^{\circ}$, $M_{Ai\max}=$ 1938.92 N·m, which was less than the maximum moment the pitch mechanism could provide in Figure 12, indicating that this pitch mechanism can meet the vehicle pitch moment requirements.

Table 5 compares simulation results with biological observation data. The ATV maximum pitch angle (26.87°) falls within the range observed during centipede obstacle crossing (25–35°), indicating good agreement between the engineering system and biological prototype. The traction force distribution pattern is consistent with centipede locomotion dynamics observed by Rieu et al. [1], validating the biomimetic design principle.

#### 4.3.3. Ground Contact Characteristic Analysis

**Figure 16:**
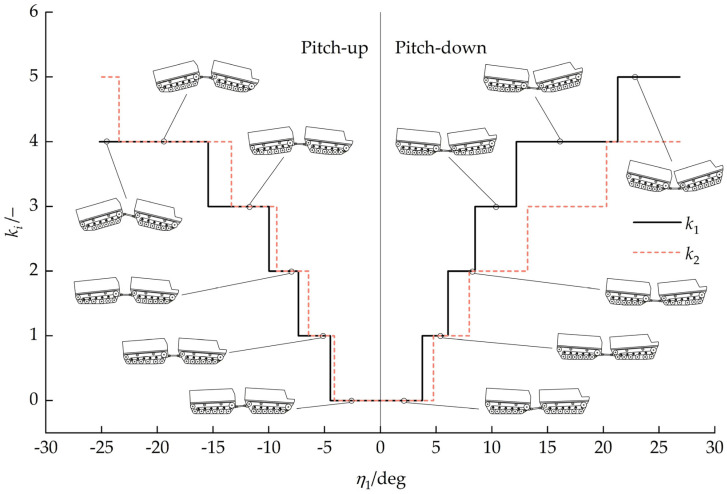
Relationship between $k_{i}$ and front-unit pitch angle $\eta_{1}$.

Figure 16 presents the relationship between the number of unloaded road wheels $k_{i}$ and the front-unit pitch angle $\eta_{1}$. With a pitch-up attitude, the road wheels at the front of the front unit and rear of the rear unit became unloaded, with the number of unloaded road wheels increasing as the front-unit pitch-up angle increased. With a pitch-down attitude, road wheels at the rear of the front unit and the front of the rear unit became unloaded, with the number of unloaded road wheels increasing as the front-unit pitch-down angle increased. When front-unit pitch angle $-4.13{}^{\circ}<\eta_{1}<3.75{}^{\circ}$, $k_{1}=k_{2}=0$, and no road wheels were unloaded.

**Figure 17:**
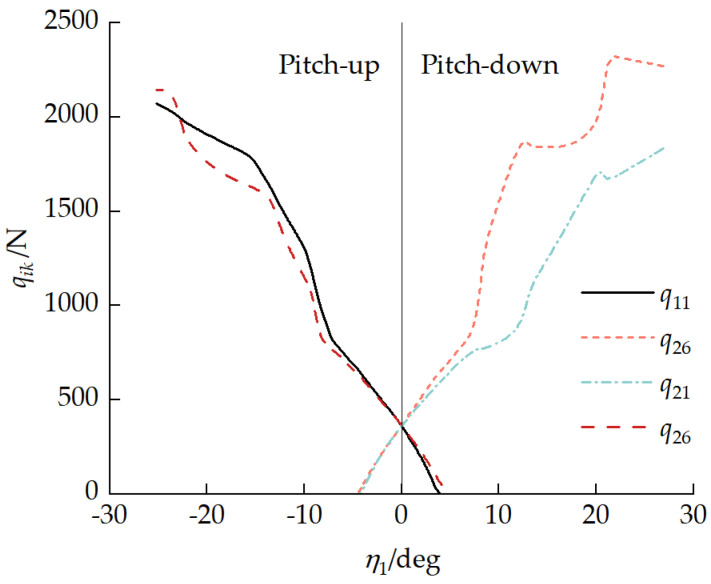
Relationships between first and last road wheel loads $q_{i1}$ and $q_{i6}$, respectively, and the front-unit pitch angle $\eta_{1}$.

Figure 17 presents the relationship between the first and last road wheel loads, $q_{i1}$ and $q_{i6}$, respectively, and the front-unit pitch angle $\eta_{1}$. With a pitch-up attitude, the last road wheel of the front unit and the first road wheel of the rear unit bore the primary vehicle load. As the front-unit pitch-up angle increased, the loads on these two road wheels increased. When the front-unit pitch angle was 7.25° and 11.91°, the rear-unit first suspension and front-unit last suspension reached their limits, respectively. With a pitch-down attitude, the first road wheel of the front unit and the last road wheel of the rear unit bore the primary vehicle load. As the front-unit pitch-down angle increased, the loads on these two road wheels increased. When the front-unit pitch angle was −7.44° and −9.29°, the front-unit first suspension and rear-unit last suspension reached their limits, respectively. The phenomenon of the front-unit suspension reaching its limit before the rear-unit suspension was mainly due to the transfer of the normal load to the front unit, as shown in Figure 13.

**Figure 18:**
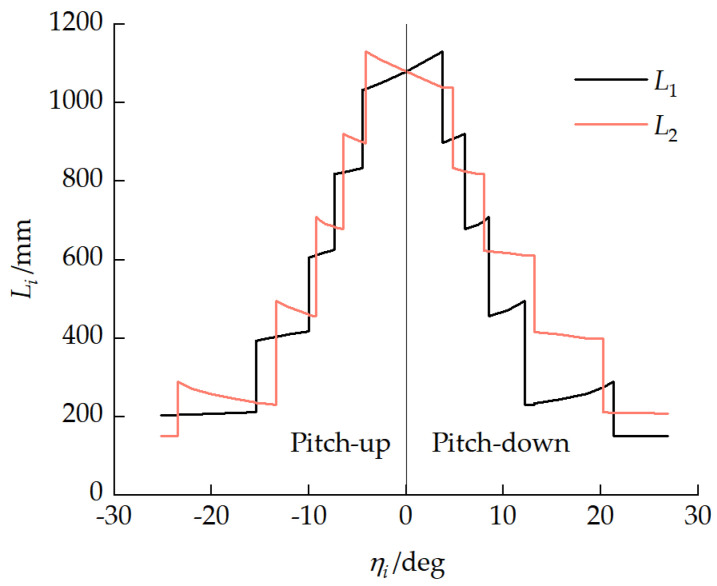
Relationship between front- and rear-unit track–ground contact lengths $L_{i}$ and front-unit pitch angle $\eta_{1}$.

Figure 18 presents the relationships between the front- and rear-unit track–ground contact lengths $L_{i}$ and front-unit pitch angle $\eta_{1}$. When the number of grounded road wheels remained unchanged, the front-unit track–ground contact length $L_{1}$ increased with increasing unit pitch-up angle $\eta_{1}$, while the rear-unit track–ground contact length $L_{2}$ decreased with increasing unit pitch-up angle $\eta_{1}$. This variation pattern was determined by the installation direction of the torsion bar suspension balance arm.

#### 4.3.4. Obstacle-Crossing Capability Analysis

**Figure 19:**
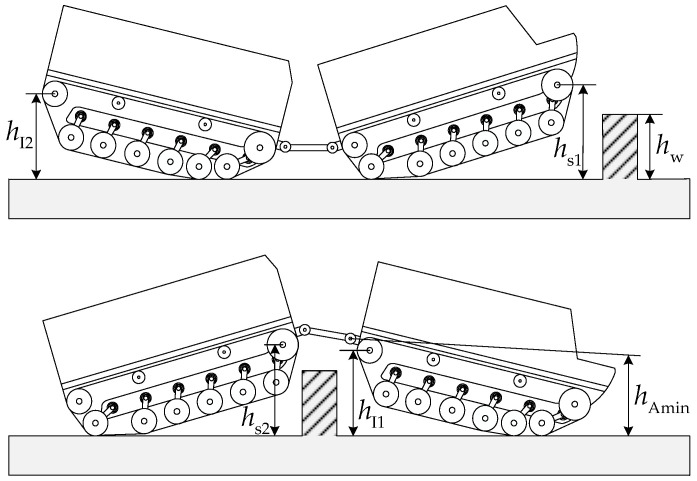
Schematic of vehicle surmounting vertical-wall obstacle.

Articulated tracked vehicles can significantly improve their terrain obstacle traversability through pitch motion. In this section, the analysis of a typical obstacle-crossing scenario with a vehicle surmounting vertical-wall obstacles is discussed. When tracked vehicles overcome vertical-wall obstacles through the static method, the surmountable vertical-wall height primarily depends on the height of the front drive sprocket center. For traditional single-unit tracked vehicles, since they cannot actively change the vehicle pitch angle significantly, the ability to overcome vertical-wall obstacles is often limited. Articulated tracked vehicles can increase the ground clearance heights of the road wheels, pitch mechanism, and other components through pitch motion, thereby increasing the surmountable vertical-wall obstacle height. Figure 19 presents the geometric constraint schematic for surmounting vertical-wall obstacles. For the articulated tracked vehicle to surmount this vertical-wall obstacle, the drive sprocket center heights $h_{si}$, idler wheel center heights $h_{Ii}$, and pitch mechanism minimum ground clearance height $h_{\mathrm{Amin}}$ must not be lower than the vertical-wall height $h_{w}$, i.e.,

(52) $$h_{w}=\min\{h_{\mathrm{Amin}},h_{si},h_{Ii}\},$$

where $h_{\mathrm{Amin}}$ is the minimum ground clearance height of the pitch mechanism, with $h_{\mathrm{Amin}}=\min\left\{h_{A1},h_{A2}\right\}$.

**Figure 20:**
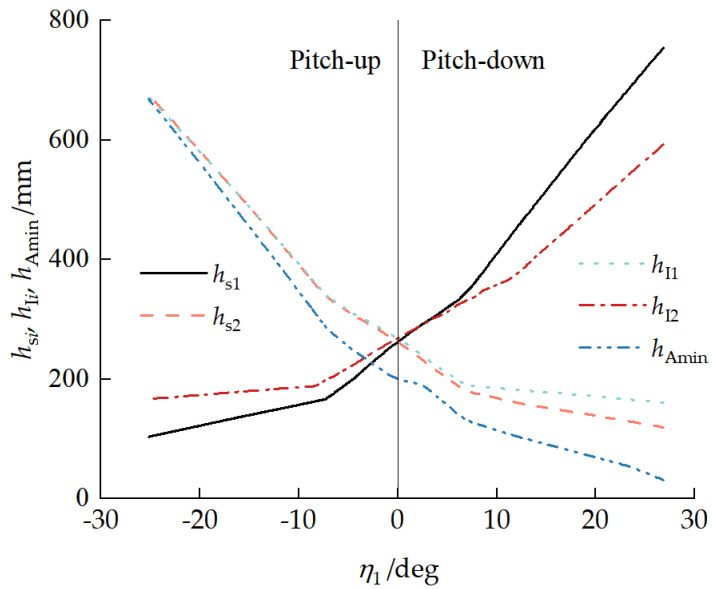
Relationship between key point heights $h_{si}$, $h_{Ii}$, and $h_{\mathrm{Amin}}$ and front-unit pitch angle $\eta_{1}$.

Figure 20 presents the relationships between key point heights $h_{si}$, $h_{Ii}$, and $h_{\mathrm{Amin}}$ and the front-unit pitch angle $\eta_{1}$. With a pitch-up attitude, as the front-unit pitch-up angle increased, the front drive sprocket center ground clearance height increased. When the front-unit pitch angle $\eta_{1}=26.87{}^{\circ}$, $h_{s1\max}$ = 754.28 mm and $h_{I2\max}$ = 592.95 mm. With a vehicle pitch-down attitude, as the front-unit pitch-down angle increased, the pitch mechanism minimum ground clearance height and rear drive sprocket center ground clearance height increased. When the front-unit pitch angle $\eta_{1}=-25.19{}^{\circ}$, $h_{s2\max}$ = 676.05 mm, $h_{I1\max}$ = 676.05 mm, and $h_{\mathrm{Amax}}$ = 668.66 mm. Therefore, the vehicle could traverse a vertical wall with a height of 592.95 mm. Compared with the initial front drive sprocket height of 263.00 mm, the vertical-wall obstacle-crossing capability was improved by 125.45%.

#### 4.3.5. Sensitivity Analysis of Suspension Articulation Torsional Stiffness

To further evaluate the robustness of the static pitch attitude adjustment, a sensitivity analysis was conducted for the suspension articulation torsional stiffness $k_{t}$. The analysis was performed for different cylinder stroke values, with the suspension stiffness varying by ±50% around its nominal value. The results, summarized in Table 6, show that the front-unit pitch angle $\eta_{1}$ remains relatively stable across different $k_{t}$ values, with only slight variations observed for different cylinder stroke lengths. For instance, at a cylinder stroke $s_{i}$ of 10 mm, the front-unit pitch angle changes from −5.02° to −4.96°, indicating minimal sensitivity to changes in $k_{t}$.

In contrast, the ground contact length $L_{1}$ is more sensitive to changes in $k_{t}$. As $k_{t}$ increases, the ground contact length $L_{1}$ decreases significantly. For example, at a cylinder stroke $s_{i}$ of 30 mm, $L_{1}$ decreases from 396.50 mm to 211.33 mm, corresponding to a reduction of approximately 47%. This reduction in ground contact length is primarily due to an increase in the number of unloaded road wheels $k_{1}$ as $k_{t}$ increases.

These findings show that while the front-unit pitch angle remains relatively insensitive to variations in articulation stiffness, the ground contact area is significantly affected by changes in $k_{t}$.

## 5. Vehicle Driving Characteristics and Cooperative Obstacle-Crossing

The pitch mechanism of articulated tracked vehicles operates in three modes: locked, compliant, and active control, with the following main characteristics.

(1) Locked mode: The cylinder stroke does not change, and there is no pitch angle between the front and rear units. The units are equivalent to a rigidly connected four-track vehicle [49], with η=0° always.
(2) Compliant mode: The cylinder stroke is in a free state. There is no constraint relationship between the front- and rear-unit pitch angles, with $M_{A1}=M_{A2}=0$ in this case.
(3) Active control mode: The cylinder stroke is changed to cause the pitch mechanism to produce a pitch angle $\eta_{Ai}$, thereby achieving the adjustment of the vehicle pitch angle η. The front- and rear-unit pitch angles satisfy $\eta =\eta_{1}-\eta_{2}$, and the pitch mechanism moments satisfy $M_{A1}=M_{A2}+\left(F_{A1z}\sin\eta_{A}+F_{A1x}\cos\eta_{A}\right)l_{A}$.

### 5.1. Constant-Velocity Driving Condition

Under active control mode, constant-velocity driving without unloaded road wheels was selected as the condition for analysis, with front-unit pitch angles $\eta_{1}$ of −3°, 0°, and 3° and the front-unit velocity $v_{1x}$ of 3 m/s. Road wheel *k* of unit *i* is denoted by the label $n_{i}\left(i-1\right)+k$. The distance from each point on the track–ground contact segment to the downward projection point of the front unit’s first road wheel center is $x_{p}$, with the following expression:

(53) $$x_{p}=\left\{\begin{array}{cc} \begin{array}{l} -x_{1}\\ l_{11}+l_{21}+l_{A}\cos\eta_{A}-x_{2} \end{array} & , \end{array}\begin{array}{c} i=1\\ i=2 \end{array}\right..$$

Changes in the vehicle pitch attitude cause variations in the road wheel loads. The logarithm of the ratio of the first to the last road wheel loads $q_{i1}/q_{in_{i}}$ in the single-side track system of unit *i* was used to describe the non-uniformity of the load distribution in that track–ground contact segment. The load non-uniformity coefficient $Q_{i}$ can be expressed as follows:

(54) $$Q_{i}=\log\frac{q_{i1}}{q_{in_{i}}}.$$

**Figure 21:**
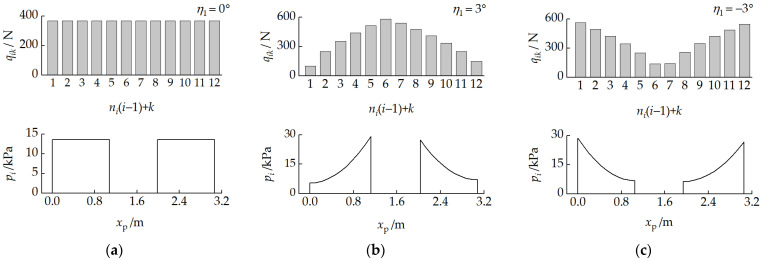
Relationship between road wheel loads $q_{ik}$ and track–ground contact pressure $p_{i}$ with different pitch attitudes: (**a**) $\eta_{1}=0{}^{\circ}$, (**b**) $\eta_{1}=3{}^{\circ}$, and (**c**) $\eta_{1}=-3{}^{\circ}$.

**Figure 22:**
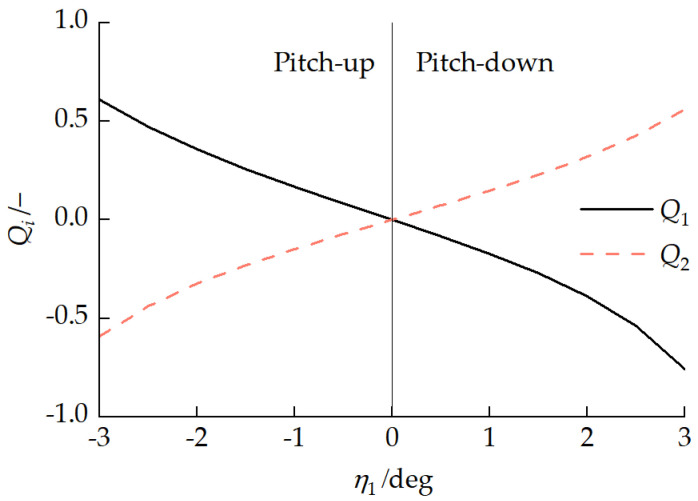
Relationship between load non-uniformity coefficient $Q_{i}$ and front vehicle body pitch angle $\eta_{1}$.

Figure 21 presents the relationship between the road wheel loads $q_{ik}$ and the track–ground contact pressure $p_{i}$ for different pitch attitudes. Figure 22 presents the relationship between the load non-uniformity coefficient $Q_{i}$ and the front-unit pitch angle $\eta_{1}$. When there was no pitch angle between the units, all road wheel loads were equal, and the front- and rear-track ground contact pressures were uniformly distributed. With a pitch-up attitude, $Q_{1}<0$, $Q_{2}>0$, and the road wheel loads and track–ground contact segment pressures near the pitch mechanism were higher. With a pitch-down attitude, $Q_{1}>0$, $Q_{2}<0$, and the road wheel loads and track–ground contact pressures away from the pitch mechanism were higher.

**Figure 23:**
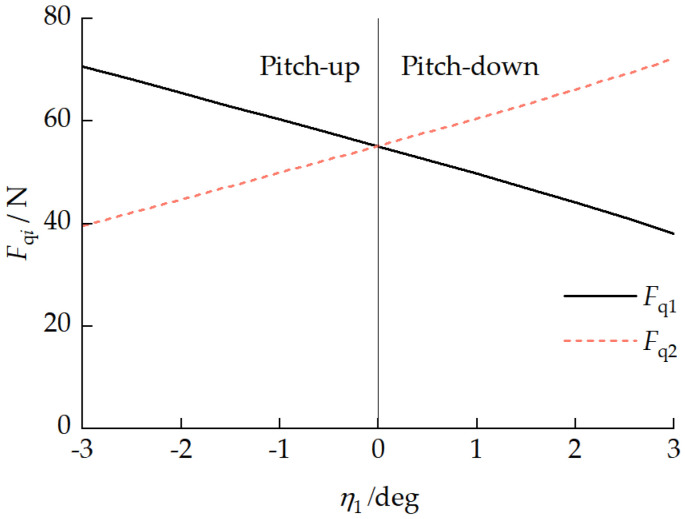
Relationship between each track traction force $F_{qi}$ and front-unit pitch angle $\eta_{1}$.

**Figure 24:**
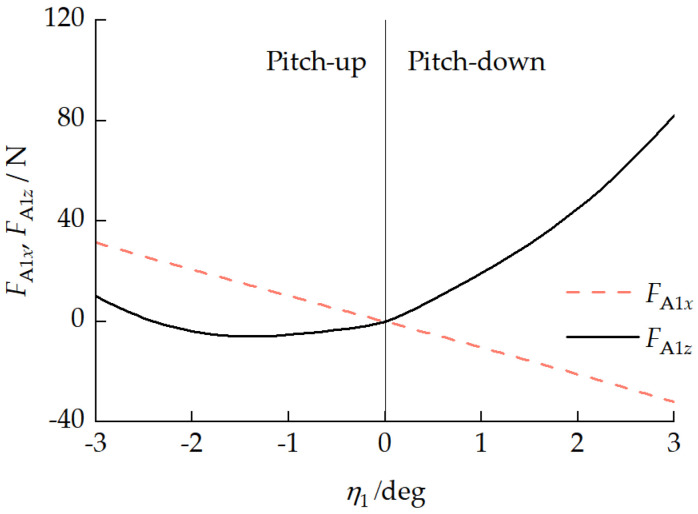
Relationship between coupling forces $F_{A1x}$ and $F_{A1z}$ and front-unit pitch angle $\eta_{1}$.

When the vehicle travels in a straight line at a constant velocity on homogeneous ground, there is no significant longitudinal interaction force between the front and rear units, and the front and rear tracks need to overcome the same ground resistance, i.e., the total traction forces of the front and rear tracks need to be consistent. However, when the vehicle has different pitch attitudes, the differences in the front- and rear-track ground contact pressure distributions lead to different traction forces for each track. Figure 23 presents the relationship between each track traction force $F_{qi}$ and the front-unit pitch angle $\eta_{1}$. Figure 24 presents the relationship between the pitch mechanism coupling forces $F_{A1x}$ and $F_{A1z}$ and the front-unit pitch angle $\eta_{1}$. With a vehicle pitch-up attitude, the front-unit track provided a greater traction force, exhibiting a “front-pulling-rear” characteristic. With a vehicle pitch-down attitude, the rear-unit track provided a greater traction force, exhibiting a “rear-pushing-front” characteristic. This traction force distribution pattern is similar to the propulsion mechanism in myriapod locomotion where the anterior segments pull and the posterior segments push. Due to the existence of a longitudinal interaction force between the front and rear units, with a vehicle pitch-up attitude, $F_{A1z}>0$, indicating the rear unit transferred a load to the front unit. With a vehicle pitch-down attitude, when the front-unit pitch angle $-2.41{}^{\circ}<\eta_{1}<0$, $F_{A1z}<0$, indicating that the front unit transferred a partial load to the rear unit.

### 5.2. Variable-Velocity Driving Condition

**Figure 25:**
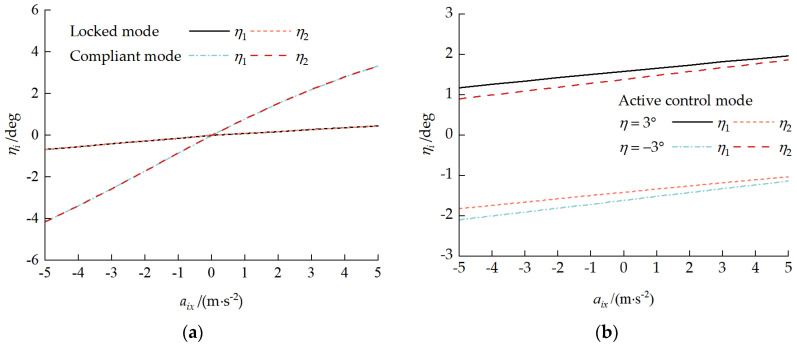
Pitch attitude response curves during variable-velocity driving: (**a**) locked and compliant modes and (**b**) active control mode.

For a front-unit velocity $v_{1x}$ = 3 m/s and an acceleration $a_{ix}$ ranging from −5 to 5 m/s^2^, the vehicle acceleration and deceleration driving characteristics under the three operating modes, locked, compliant, and active control, were analyzed. Figure 25 presents the pitch attitude response curves during variable-velocity driving. Under locked and compliant modes, the inter-unit pitch angle η=0°. During acceleration, each unit assumed a pitch-up attitude; during deceleration, each unit assumed a pitch-down attitude. The larger the absolute value of the acceleration was, the more significant the pitch angle change was, and this change was more pronounced in compliant mode. Under active control mode, the inter-unit pitch angle maintained the set value of η=±3°. During acceleration, each unit pitch angle increased; during deceleration, each unit pitch angle decreased.

**Figure 26:**
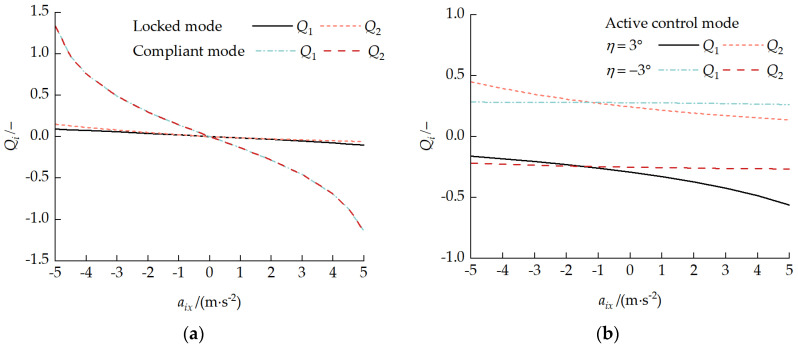
Load distribution characteristic curves during variable-velocity driving: (**a**) locked and compliant modes and (**b**) active control mode.

Figure 26 presents the load distribution characteristic curves during variable-velocity driving. As shown in Figure 26a, under locked and compliant modes, when the acceleration was zero, the track–ground contact pressure was uniformly distributed. During acceleration, the rear road wheel loads increased, and the non-uniformity coefficient $Q_{i}$ change was more dramatic in compliant mode. As shown in Figure 26b, under active control mode with a pitch-up attitude at a pitch angle of 3°, the acceleration exacerbated the front unit’s load non-uniformity while weakening the rear-unit non-uniformity. With a pitch-down attitude at a pitch angle of −3°, the effect of acceleration changes on the load distribution non-uniformity was not significant, mainly because the changes in each unit pitch angle shown in Figure 25b offset the effect of the acceleration.

**Figure 27:**
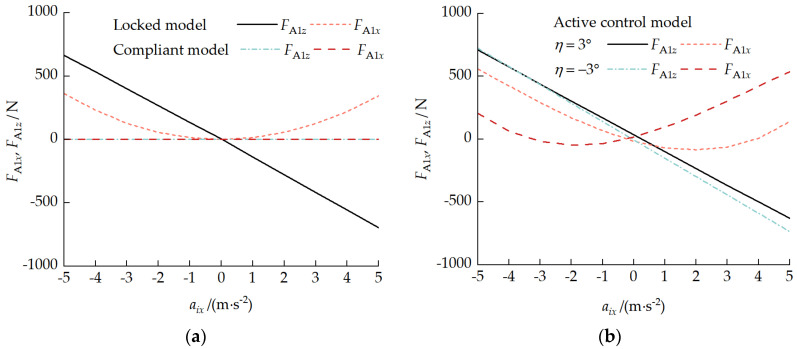
Coupling force characteristic curves during variable-velocity driving: (**a**) locked and compliant modes and (**b**) active control mode.

Figure 27 presents the coupling force characteristic curves during variable-velocity driving. In compliant mode, there was no significant interaction force between the front and rear units. In locked mode, $F_{A1x}\geq 0$, with $a_{ix}=0$ as the turning point. During the deceleration phase, the front unit dominated the braking, while during the acceleration phase, the rear unit dominated the driving. The overall characteristic was rear-pushing-front. Under active control mode, with a pitch-up attitude when η=3° and −0.22 m/s^2^ $\leq a_{ix}\leq$ 3.93 m/s^2^ and with a pitch-down attitude when η=−3° and −3.27 m/s^2^ $\leq a_{ix}\leq$ −0.26 m/s^2^, $F_{A1x}<0$, and a front-pulling-rear characteristic was exhibited. Under the other conditions, a rear-pushing-front characteristic was exhibited.

### 5.3. Cooperative Obstacle-Crossing Method

Myriapods employ wave-like inter-segment coordination strategies during obstacle crossing: the anterior segments first lift to traverse obstacles, followed by successive segments, forming a temporally coordinated motion sequence. Inspired by this, this section proposes a cooperative obstacle-crossing motion planning method for articulated tracked vehicles. By reasonably planning the pitch timing and magnitude of the front and rear bodies, efficient obstacle crossing is achieved. The process of articulated tracked vehicles overcoming terrain obstacles is a dynamic and continuous cooperative motion process that requires coordination between the track drive system and the pitch mechanism. Based on the established pitch motion dynamics model, this section proposes a vehicle attitude prediction method and a cooperative obstacle-crossing motion planning framework.

#### 5.3.1. Motion Planning Framework

When the vehicle overcomes terrain obstacles such as vertical walls and trenches, it needs to adjust the pitch attitude within a specified time to meet the geometric traversability requirements. Cooperative obstacle-crossing motion planning for multiple vehicle actuators needs to consider spatiotemporal and dynamic constraints, mainly including the following steps.

(1) Obstacle-crossing action sequence generation

Based on the geometric features of terrain obstacles, with vehicle longitudinal displacement $X_{j}$ as the independent variable, the pitch attitude sequence required for collision-free vehicle passage is calculated. With the process of surmounting a vertical-wall obstacle of height $h_{w}$ at a constant velocity as an example, the action sequence needs to satisfy the following geometric traversability constraints: at each key position, both the drive sprocket center height and the pitch mechanism lowest point height must not be lower than the obstacle height. The action sequence can be expressed as a collection of discrete path points:

(55) $$P=\left\{\left.\left(X_{j},\eta_{1,j},\eta_{2,j}\right)\right|j=1,2,\ldots ,N\right\},$$

where *N* is the number of path points, and each path point defines the front- and rear-unit pitch angles $\eta_{1,j}$ and $\eta_{2,j}$ that the vehicle should achieve at the longitudinal displacement $X_{j}$.

(2) Trajectory time parameterization

Let the specified time for obstacle-crossing be *T* and the initial vehicle velocity be $v_{0x}$. Time stamps are assigned to the geometric path points to convert static paths into time-varying trajectories:

(56) $$T=\left\{\left.\left(t_{j},X_{j},\eta_{1,j},\eta_{2,j}\right)\right|j=1,2,\ldots ,N\right\}.$$

Adjacent trajectory points need to satisfy the following motion differential constraints:

(57) $$\left\{\begin{array}{l} X_{j+1}-X_{j}={\bar{v}}_{j}\left(t_{j+1}-t_{j}\right)\\ \eta_{i\left(j+1\right)}-\eta_{ij}={\bar{\dot{\eta }}}_{j}\left(t_{j+1}-t_{j}\right) \end{array}\right.,$$

where ${\bar{v}}_{j}$ and ${\bar{\dot{\eta }}}_{j}$ are the average vehicle velocity and average pitch angular velocity during this time period, respectively.

(3) Feasible trajectory constraint conditions

The actuators need to satisfy the following physical constraint conditions for the reference trajectory:

• Cylinder stroke constraint: $s_{i,\min}<s_{i}<s_{i,\max}$.
• Cylinder extension/retraction velocity constraint: ${\dot{s}}_{i}<{\dot{s}}_{i,\max}$.
• Maximum working pitch moment constraint: $\left|M_{Ai}\right|<\left|M_{oAi}\right|$.
• Drive sprocket angular acceleration constraint: $\left|{\dot{\omega }}_{i}\right|={\dot{\omega }}_{i,\max}$.

(4) Cooperative motion inverse solution

With the optimized trajectory as the input, an inverse solution is obtained through the pitch motion dynamics model to calculate the motion state of each actuator at each moment. Let the desired state at time *t* be $\left(X\left(t\right),\eta_{1}\left(t\right),\eta_{2}\left(t\right),{\dot{\eta }}_{1}\left(t\right),{\dot{\eta }}_{2}\left(t\right)\right)$. The inverse solution outputs are the pitch cylinder stroke $s_{i}$, track drive sprocket angular velocity ${\dot{\omega }}_{i}$, and pitch cylinder force ${\dot{\omega }}_{i}$.

(5) Command generation and execution

The motion state sequence obtained from the inverse solution is converted into control commands for the actuators, including hydraulic valve flow commands and motor speed or current commands. The lower-level controller receives commands and drives the actuators to complete the cooperative pitch obstacle-crossing motion.

#### 5.3.2. Obstacle-Crossing Case Validation

To validate the effectiveness of the proposed cooperative obstacle-crossing motion planning method, the articulated tracked vehicle surmounting a typical vertical-wall obstacle was selected as an example for simulation analysis. The vertical-wall obstacle parameters were set as follows: height $h_{w}=400\;\mathrm{mm}$ and width $b_{w}=100\;\mathrm{mm}$. The vehicle initial position was 1000 mm from the vertical wall at the front-track leading edge of the front unit to the vertical wall. Obstacle-crossing planning was performed with the front-unit longitudinal velocity $v_{1x0}=0.5\;m/s$ as the reference velocity.

**Figure 28:**
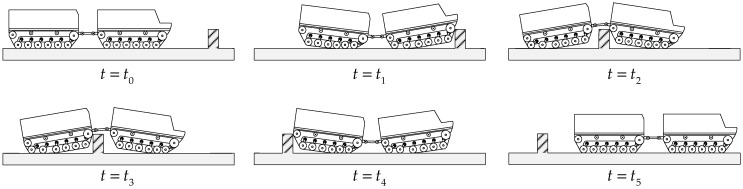
Phase division for vehicle surmounting vertical-wall obstacle.

Based on the geometric contact relationship between the vehicle and obstacle, the obstacle-crossing process was divided into five phases, as shown in Figure 28.

• Phase 1: Approach phase ($t_{0}<t\leq t_{1}$). The vehicle moved from its initial position until the front-unit track contacted the vertical-wall outer corner line. The vehicle transitioned from a horizontal state to a pitch-up attitude. The geometric constraint condition for this phase was that the front drive sprocket center was higher than the vertical wall $h_{s11}\geq h_{w}$.
• Phase 2: Front-unit obstacle-crossing phase ($t_{1}<t\leq t_{2}$). The front unit passed over the vertical-wall obstacle, from the front-unit track contacting the vertical-wall same-side outer corner line until the front-unit rear track left the vertical-wall opposite-side outer corner line. The vehicle transitioned from a pitch-up attitude to a pitch-down attitude. The geometric constraint condition for this phase was that the front idler wheel center was higher than the vertical wall $h_{I12}\geq h_{w}$.
• Phase 3: Transition phase ($t_{2}<t\leq t_{3}$). During this period, the pitch mechanism passed over the vertical wall, from the front-unit track leaving the vertical-wall opposite-side outer corner line until the rear-unit track contacted the same-side outer corner line. The vehicle maintained a pitch-down attitude. The geometric constraint condition for this phase was that the pitch mechanism’s lowest ground clearance point was higher than the vertical wall $h_{A\min}\geq h_{w}$.
• Phase 4: Rear-unit obstacle-crossing phase ($t_{3}<t\leq t_{4}$). The rear unit passed over the vertical-wall obstacle, from the rear-unit track contacting the vertical-wall same-side diagonal line until the rear-unit track left the vertical-wall opposite-side outer corner line. The vehicle transitioned from a pitch-down attitude to a pitch-up attitude. The geometric constraint condition for this phase was that the rear idler wheel center was higher than the vertical wall $h_{I24}\geq h_{w}$.
• Phase 5: Recovery phase ($t_{4}<t\leq t_{5}$). The vehicle recovered from the pitch-up attitude to a horizontal state.

Based on the above geometric traversability constraints and the data in Figure 19, the key state parameters for each phase were solved for. Table 7 summarizes the geometric constraint parameters for each phase, including the key heights, pitch angles, and cylinder strokes.

Based on reference velocity $v_{1x0}$ = 0.5 m/s and the geometric relationships of each phase, the calculated durations of each phase were $t_{1}=2\;s$, $t_{2}=3.02\;s$, $t_{3}=0.73\;s$, $t_{4}=3.17\;s$, and $t_{5}=1\;s$. The total obstacle-crossing time was 9.92 s. Furthermore, based on the dynamics model inverse solution, the drive sprocket angular velocities at each key moment were obtained, as shown in Table 8.

To achieve continuous and executable obstacle-crossing control, the above discrete state points needed to be connected into smooth reference trajectory curves. The Piecewise Cubic Hermite Interpolating Polynomial (PCHIP) method was adopted for trajectory planning, which ensured monotonicity preservation and C1 continuity, thereby avoiding non-physical overshoot or oscillations while satisfying the kinematic smoothness requirements of the actuators.

Let the key state point sequence be $\left\{\left(t_{j},y_{j}\right)\right\}$, *j* = 0, 1, …, 5, where $y_{j}$ represents state parameters such as the cylinder stroke $s_{i}$ or the drive sprocket angular velocity $\omega_{i}$. In each segment interval $\left[t_{j},t_{j+1}\right]$, Hermite basis functions were used for interpolation, and node derivatives were determined by weighted harmonic averaging of adjacent difference quotients to ensure curve monotonicity.

**Figure 29:**
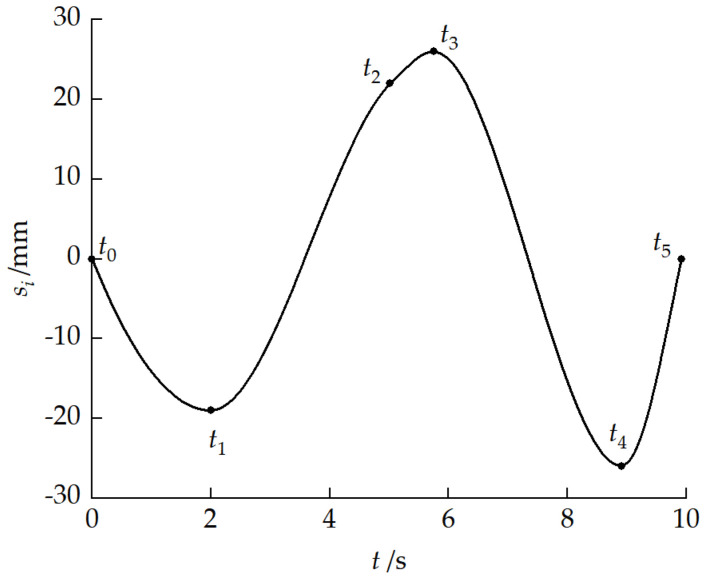
Cylinder stroke planning curve.

Figure 29 presents the pitch cylinder stroke planning curve. In the approach phase, the cylinder retracted to −19 mm to achieve a pitch-up attitude. In the front-unit obstacle-crossing phase, the cylinder extended to 22–26 mm to achieve a pitch-down attitude. In the rear-unit obstacle-crossing phase, the cylinder retracted again to −26 mm. Finally, in the recovery phase, it returned to its initial position.

**Figure 30:**
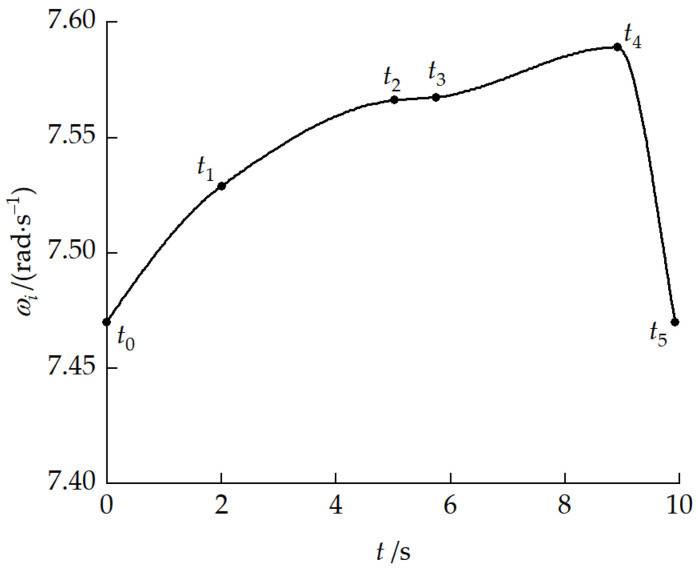
Drive sprocket angular velocity planning curve.

Figure 30 presents the drive sprocket angular velocity planning curve. During the obstacle-crossing process, pitch angular velocity changes occurred between the front and rear units, and longitudinal velocity differences existed when the two units were at different height planes. The drive sprocket angular velocity adaptively adjusted within the range of 7.47–7.59 rad/s to compensate for the kinematic coupling effects caused by pitch attitude changes, ensuring smooth obstacle-crossing.

The calculation results demonstrated that the established dynamics model and cooperative motion planning method can effectively handle the obstacle-crossing motion planning problem of articulated tracked vehicles, providing a reliable theoretical basis for obstacle-crossing control system design.

#### 5.3.3. Comparison with Commercial Multibody Simulation

To validate the proposed pitch motion model, a commercial multibody dynamics simulation was conducted using RecurDyn V9R4, which is widely adopted for high-fidelity simulation of tracked vehicles. A virtual prototype of the articulated tracked vehicle was established with the same structural configuration and key parameters as those used in the theoretical model.

**Figure 31:**
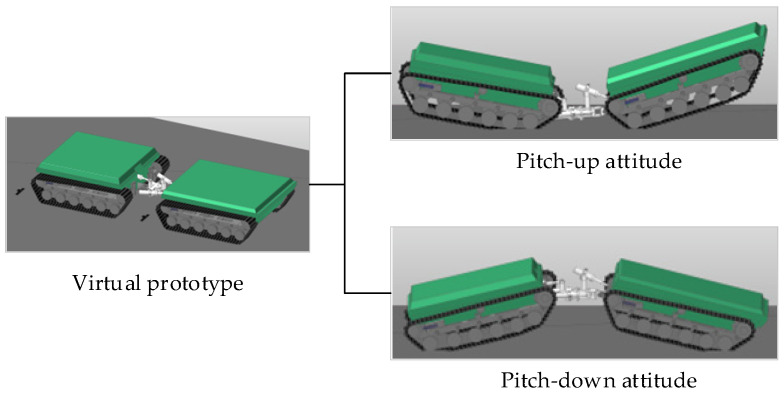
Virtual prototype of the articulated tracked vehicle and its simulated pitch-up and pitch-down attitudes.

Figure 31 shows the virtual prototype and the simulated pitch-up and pitch-down attitudes during the obstacle-crossing process. The simulation reproduces the coordinated pitch motion between the front and rear units predicted by the proposed model.

**Figure 32:**
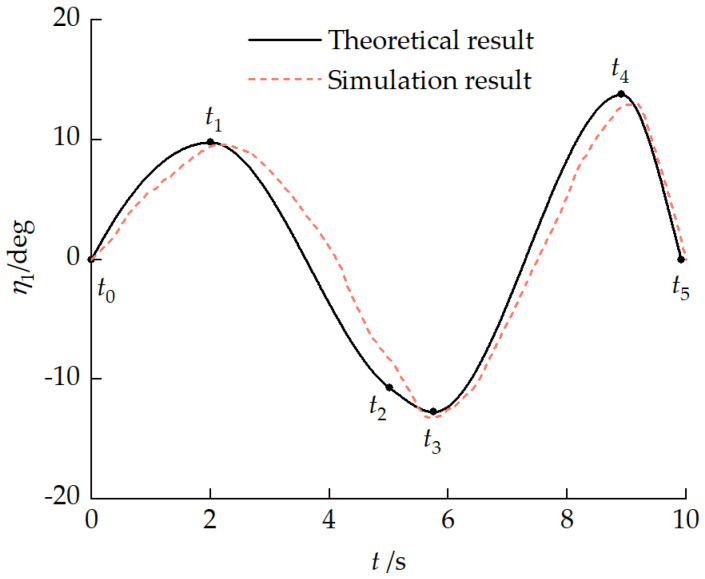
Comparison of the front-unit pitch angle responses predicted by the theoretical model and the multibody simulation during obstacle crossing.

Figure 32 compares the time histories of the front-unit pitch angle obtained from the theoretical model and the RecurDyn simulation during obstacle crossing. The two results exhibit good agreement in terms of overall trend, phase evolution, and peak pitch angles. Minor discrepancies are observed near the extreme pitch positions, which can be attributed to the additional compliance and contact nonlinearities captured by the multibody simulation but simplified in the theoretical model.

These results demonstrate that the proposed pitch motion model can effectively capture the essential dynamic characteristics of the articulated tracked vehicle during obstacle crossing, thereby providing a reasonable and reliable basis for subsequent analysis.

### 5.4. Energy-Saving Implications of Biomimetic Pitch Adjustment

The proposed biomimetic pitch adjustment strategy, although primarily developed for dynamic modeling and traction analysis, also implies potential energy-saving benefits during obstacle-crossing maneuvers. By actively regulating the pitch attitude, the track–ground contact pressure is more evenly distributed under obstacle-crossing conditions, thereby reducing excessive local load concentration and unloading of individual road wheels. As a result, available traction can be utilized more effectively, and energy losses associated with track slip and repeated contact–separation events are mitigated.

Furthermore, cooperative traction allocation between the front and rear units enables a more balanced distribution of driving effort under varying pitch conditions. Compared with passive pitch response or fixed traction allocation strategies, the proposed approach limits redundant torque output from individual units and improves overall traction efficiency during obstacle negotiation. From a mechanical perspective, this coordinated driving behavior reduces ineffective work input and enhances energy utilization, particularly in complex terrain environments.

A quantitative assessment of energy consumption would require detailed powertrain modeling and efficiency characterization, which are beyond the scope of the present study. Nevertheless, the dynamic and traction analysis results presented herein provide a clear mechanistic basis for future investigations into energy-efficient motion planning and control strategies for articulated tracked vehicles.

## 6. Conclusions

Inspired by myriapod locomotion mechanisms, this study established a rigid–flexible coupled dynamics model for articulated tracked vehicle pitch motion and systematically analyzed the influence of pitch attitude on vehicle dynamics and obstacle-crossing capability. The main conclusions are as follows:

(1) A pitch kinematics model incorporating torsion bar suspension stiffness was established, revealing coupling relationships among unit pitch angle, suspension deformation, and track–ground contact length. Under pitch-up conditions, front-unit ground contact length increases while rear-unit length decreases; the opposite occurs under pitch-down conditions. This pattern is governed by suspension balance arm orientation.

(2) A quadratic track–ground contact pressure distribution method was proposed. This method uses road wheel vertical loads as constraints and determines pressure distribution coefficients through moment equilibrium, overcoming the non-physical negative pressure issue of traditional linear assumptions under large pitch angles.

(3) The influence of pitch attitude on front–rear traction force allocation was revealed. Under pitch-up conditions, the front unit provides primary traction in a front-pulling-rear driving mode; under pitch-down and acceleration conditions, the rear unit dominates in a rear-pushing-front driving mode. This pattern exhibits consistency with the anterior-guidance and posterior-propulsion mechanism observed in centipede locomotion, reflecting the net mechanical effect of distributed segment coordination at a functional level.

(4) Through pitch attitude adjustment, the maximum surmountable vertical-wall height increased from 263 to 593 mm, representing a 125% improvement. The maximum pitch angle of 26.87° falls within the biological range of 25–35° observed during centipede obstacle crossing, validating the biomimetic design approach in terms of functional-scale consistency rather than morphology-level replication.

(5) A cooperative obstacle-crossing motion planning framework was constructed, generating smooth actuator trajectories through Piecewise Cubic Hermite interpolation. The 400 mm vertical-wall crossing case validated method feasibility.

It should be emphasized that the two-unit “front-pulling–rear-pushing” mode identified in this study represents an engineering-level functional abstraction of myriapod locomotion, rather than a direct reproduction of multi-segment wave propagation mechanisms. This abstraction captures the dominant mechanical effect of distributed segment coordination, which is characterized by the functional separation between anterior guidance and posterior propulsion and underlies the traction allocation patterns and pitch regulation behaviors discussed in this study. At the same time, this simplification inevitably neglects higher-order biological features, including phase-lagged wave propagation across multiple segments, local compliance modulation, and neural feedback-driven coordination, which fall beyond the scope of the present engineering-oriented model. While these mechanisms contribute to adaptability and robustness in biological systems, they are difficult to exploit directly in articulated tracked vehicles with limited degrees of freedom. Therefore, the proposed two-unit abstraction represents a deliberate trade-off between biological fidelity and engineering feasibility, providing a rational and tractable framework for dynamics modeling and obstacle-crossing motion planning, while leaving more detailed bio-inspired coordination strategies as potential directions for future research.

## Figures and Tables

**Table 1:** Structure–function correspondence at the level of functional abstraction between myriapods and ATVs.

Biological Structure	Engineering Structure	Functional Correspondence	Characteristic Parameters
Inter-segment dorsoventral joint	Hydraulic pitch mechanism	Active pitch adjustment	Pitch angle ±25–35°
Leg base compliant structure	Torsion bar spring suspension	Passive terrain adaptation	Passive adaptation
Multi-leg distributed contact	Multi-road-wheel track system	Load distribution and traction	Distributed ground contact
Anterior–posterior segment coordination	Front–rear unit coupled drive	Coordinated propulsion mode	Sequential activation

**Table 2:** Key point coordinates for vehicle pitch attitude.

Symbol	Coordinates
$p_{i1}$	$\left(r_{b}\cos\theta_{i1},\;r_{b}\sin\theta_{i1}\right)$
$o_{si}$	$\left(r_{b}\cos\theta_{i1}+l_{si}\cos\left(\beta_{si}+\eta_{i}\right),\;r_{b}\sin\theta_{i1}+l_{si}\sin\left(\beta_{si}+\eta_{i}\right)\right)$
$o_{Ii}$	$\left(r_{b}\cos\theta_{i1}-l_{pi}\cos\eta_{i}-l_{Ii}\cos\left(\beta_{Ii}-\eta_{i}\right),\;r_{b}\sin\theta_{i1}+l_{pi}\sin\eta_{i}+l_{Ii}\sin\left(\beta_{Ii}-\eta_{i}\right)\right)$
$o_{i}$	$\left(r_{b}\cos\theta_{i1}-l_{oi}\cos\left(\beta_{oi}-\eta_{i}\right),\;r_{b}\sin\theta_{i1}+l_{oi}\sin\left(\beta_{oi}-\eta_{i}\right)\right)$
$p_{ik}$	$\left(r_{b}\cos\theta_{i1}-l_{pi}\frac{k-1}{n_{i}-1}\cos\eta_{i},\;r_{b}\sin\theta_{i1}-l_{pi}\frac{k-1}{n_{i}-1}\sin\eta_{i}\right)$
$o_{ik}$	$\left(r_{b}\left(\cos\theta_{i1}-\cos\theta_{ik}\right)-l_{pi}\frac{k-1}{n_{i}-1}\cos\eta_{i},\;r_{b}\left(\sin\theta_{i1}-\sin\theta_{ik}\right)-l_{pi}\frac{k-1}{n_{i}-1}\sin\eta_{i}\right)$
$p_{in_{i}}$	$\left(r_{b}\cos\theta_{i1}-l_{pi}\cos\eta_{i},\;r_{b}\sin\theta_{i1}-l_{pi}\sin\eta_{i}\right)$
$o_{Ai}$	$\left(r_{b}\cos\theta_{i1}-l_{pi}\cos\eta_{i}-l_{Ai}\cos\left(\beta_{Ai}-\eta_{i}\right),\;r_{b}\sin\theta_{i1}-l_{pi}\sin\eta_{i}+l_{Ai}\sin\left(\beta_{Ai}-\eta_{i}\right)\right)$

**Table 3:** Main parameters of the articulated tracked vehicle and the ground.

Parameters	Value	Parameters	Value
$m_{i}$/kg	450	$I_{si}$ /(kg·m^2^)	0.0056
$I_{iy}$/(kg·m^2^)	65.7	$I_{w}$/(kg·m^2^)	0.011
$L_{i0}$/mm	1080	$l_{10}$/mm	765.5
b/mm	230	$l_{20}$/mm	849.5
$\beta_{si}$/deg	56.07	$l_{A}$/mm	370.73
$l_{si}$/mm	100	$l_{oi}$/mm	678.75
$\beta_{Ii}$/deg	25.13	$\beta_{oi}$/deg	17.31
$l_{Ii}$/mm	209.5	$l_{A1}$/mm	333.88
$l_{pi}$/mm	1080	$l_{A2}$/mm	202.13
$n_{i}$/−	6	$\beta_{A1}$/deg	2.75
$k_{t}$/(N·m/deg)	123	$\beta_{A2}$/deg	4.54
$\theta_{s}$/deg	33.52	$f_{0}$/−	0.03
$r_{b}$/mm	130.5	$f_{r}$/−	0.025
$r_{d}$/mm	67.5	$f_{s}$/−	0.00015
r/mm	75	μ/−	0.68
$h_{t}$/mm	33	K */−*	0.12

**Table 4:** Main parameters of the pitch mechanism.

Parameters	Value	Parameters	Value
$s_{1}$/mm	205.82–305.82	$c_{2}$/mm	261.7
$s_{2}$/mm	207.51–307.51	$c_{3}$/mm	125.1
$\phi_{A1}$/deg	73.42°	$c_{4}$/mm	295.86
$\phi_{A3}$/deg	60.13°	$p_{c}$/MPa	16
$c_{1}$/mm	125	$e_{c}$/−	0.9

**Table 5:** Comparison of simulation results with biological prototype parameters.

Parameter	Biological Prototype (Centipede)	ATV Simulation Results
Maximum pitch angle	25–35° [2,3]	26.87°
Traction distribution	Anterior-pull posterior-push [1]	Front-pull rear-push
Coordination mode	Wave-like segment Coordination [5]	Sequential phase coordination

**Table 6:** Sensitivity analysis of $\eta_{1}$, $L_{1}$, and $k_{1}$ with varying $k_{t}$.

$s_{i}$/mm	$\eta_{1}$/deg			$L_{1}$/mm			$k_{1}$/−		
	$0.5k_{t}$	$k_{t}$	$1.5k_{t}$	$0.5k_{t}$	$k_{t}$	$1.5k_{t}$	$0.5k_{t}$	$k_{t}$	$1.5k_{t}$
10	−5.02	−5.00	−4.96	1026.97	830.73	626.26	0	1	2
20	−9.76	−9.76	−9.76	606.52	606.52	417.18	2	2	3
30	−14.77	−14.77	−14.77	396.50	396.96	211.33	3	3	4

**Table 7:** Key state parameters for each phase.

Phase	Height	$\eta_{1}$/deg	$\eta_{2}$/deg	$s_{i}$/mm
1	$h_{s1}=404.65$	9.77	−7.86	−19
2	$h_{I1}=407.72$	−10.76	9.83	22
3	$h_{\mathrm{Amin}}=406.83$	−12.75	11.65	26
4	$h_{I2}=402.35$	13.77	−10.41	−26

**Table 8:** Drive sprocket angular velocities at key moments.

Moment	$t_{0}$	$t_{1}$	$t_{2}$	$t_{3}$	$t_{4}$	$t_{5}$
$\omega_{i}$/$\mathrm{rad}\cdot s^{-1}$	7.47	7.53	7.56	7.57	7.59	7.47

## Data Availability

Data are contained within the article.
